# Dense Multi-Scale Graph Convolutional Network for Knee Joint Cartilage Segmentation

**DOI:** 10.3390/bioengineering11030278

**Published:** 2024-03-14

**Authors:** Christos Chadoulos, Dimitrios Tsaopoulos, Andreas Symeonidis, Serafeim Moustakidis, John Theocharis

**Affiliations:** 1 Department of Electrical & Computer Engineering, Aristotle University of Thessaloniki, 54124 Thessaloniki, Greece; christgc@auth.gr (C.C.); symeonid@ece.auth.gr (A.S.); s.moustakidis@aideas.eu (S.M.); 2 Institute for Bio-Economy and Agri-Technology, Centre for Research and Technology—Hellas, 38333 Volos, Greece; d.tsaopoulos@certh.gr

**Keywords:** knee cartilage osteoarthritis (*KOA*), magnetic resonance imaging (*MRI*) segmentation, multi-atlas, graph neural networks (*GNNs*), deep learning, graph learning, semi-supervised learning (*SSL*)

## Abstract

In this paper, we propose a dense multi-scale adaptive graph convolutional network (*DMA-GCN*) method for automatic segmentation of the knee joint cartilage from MR images. Under the multi-atlas setting, the suggested approach exhibits several novelties, as described in the following. First, our models integrate both local-level and global-level learning simultaneously. The local learning task aggregates spatial contextual information from aligned spatial neighborhoods of nodes, at multiple scales, while global learning explores pairwise affinities between nodes, located globally at different positions in the image. We propose two different structures of building models, whereby the local and global convolutional units are combined by following an alternating or a sequential manner. Secondly, based on the previous models, we develop the *DMA-GCN* network, by utilizing a densely connected architecture with residual skip connections. This is a deeper *GCN* structure, expanded over different block layers, thus being capable of providing more expressive node feature representations. Third, all units pertaining to the overall network are equipped with their individual adaptive graph learning mechanism, which allows the graph structures to be automatically learned during training. The proposed cartilage segmentation method is evaluated on the entire publicly available Osteoarthritis Initiative (*OAI*) cohort. To this end, we have devised a thorough experimental setup, with the goal of investigating the effect of several factors of our approach on the classification rates. Furthermore, we present exhaustive comparative results, considering traditional existing methods, six deep learning segmentation methods, and seven graph-based convolution methods, including the currently most representative models from this field. The obtained results demonstrate that the *DMA-GCN* outperforms all competing methods across all evaluation measures, providing DSC=95.71% and DSC=94.02% for the segmentation of femoral and tibial cartilage, respectively.

## 1. Introduction

Osteoarthritis (OA) is one of the most prevalent joint diseases worldwide, causing pain and mobility issues, reducing the ability to lead an independent lifestyle, and ultimately decreasing the quality of life in patients. It primarily manifests among populations of advanced age, with an estimated 10% of people over the age of 55 dealing with this condition. That percentage is likely to noticeably increase in the coming years, especially in the developing parts of the world where life expectancy is steadily on the rise [1].

Among the available imaging modalities, magnetic resonance imaging (MRI) constitutes a valuable tool in the characterization of the knee joint, providing a robust quantitative and qualitative analysis for detecting anatomical changes and defects in the cartilage tissue. Unfortunately, manual delineations performed by human experts are resource- and time-consuming, while also suffering from unacceptable levels of inter- and intrarater variability. Thus, there is an increasing demand for accurate and time-efficient fully automated methods for achieving reliable segmentation results.

During the past decades, a considerable amount of research has been conducted to achieve the above goals. However, the thin cartilage structure, as well as the great variability in and intensity inhomogeneity of MR images have posed significant challenges. Several methods are proposed to address those issues, ranging from more traditional image processing ones such as statistical shape models and active appearance models, to more automated ones employing classical machine learning and deep learning techniques. A comprehensive review of such automated methods for knee articular segmentation can be found in [2].

### 1.1. Statistical Shape Methods

A wide variety of methods that fall under the statistical shape model (*SSM*) and active appearance model (*AAM*) family have been extensively employed in knee joint segmentation applications in the past. Since the specific shape of the cartilage structure is quite distinct and characteristic, these methods employ this feature as a stepping stone towards complete delineation for the whole knee joint [3]. Additionally, *SSMs* have been successfully utilized as shape regularizers within more complex segmentation pipelines, mainly as a final postprocessing step [4]. While conceptually simple, these methods are highly sensitive to the initial landmark selection process.

### 1.2. Machine Learning Methods

Under the classical machine learning setting, knee cartilage segmentation is cast as a supervised classification task, estimating the label of each voxel from a set of handcrafted or automatically extracted features from the available set of images. Typical examples of such approaches can be found in [5,6]. These methods are conceptually simple but usually offer mediocre results, due to their poor generalization capabilities and the utilization of fixed feature descriptors that may not be well suited to efficiently capture the data variability.

### 1.3. Multi-Atlas Patch-Based Segmentation Methods

Multi-atlas patch-based methods have long been a staple in medical imaging applications [7,8]. Utilizing an atlas library $A={\left\{A_{i},L_{i}\right\}}_{i=1}^{n_{A}}$ comprising $n_{A}$ magnetic resonance images $\left(A_{i}\right)$ and their corresponding label maps $L_{i}$, these methods operate on a single target image T at a time, annotating it by propagating voxel labels from the atlas library A. The implicit assumption in this framework is that the target image T and the corresponding images comprising the atlas library A reside in a common coordinate space. This assumption is enforced by registering all atlases $A_{i}\in A$ along with their corresponding labels maps $L_{i}$ to the target image space, via an affine or deformable transformation [9].

Multi-atlas patch-based methods usually consist of the following steps: For all voxels x∈T, a search volume N(x) of size $N_{s}=\left(n\times n\times n\right)$ centered around x is formed, and every corresponding voxel y∈N(x) in the spatially adjacent locations in the registered atlases yields a patch library $P_{L}=p_{A_{i}}\left(y\right),\forall y\in N\left(x\right)$ for all atlases $i=1,\cdots ,n_{A}$. An optimization problem such as sparse coding (*SC*) is then used to reconstruct the target patch as a linear combination of its corresponding atlas library. Established methods of this category of segmentation algorithms are presented in [7,8]. Despite being capable of achieving appreciable results, these methods do not scale well to large datasets due to the intense computational demands of constructing a patch library and solving an optimization problem for each voxel of every target image.

### 1.4. Deep Learning Methods

The recent resurgence of deep learning has had a great impact on medical imaging applications, with an increasing number of works reporting the use of deep architectures in various applications pertaining to that field. Initially restricted to 2D models due to the large computational load imposed by the 3D structure of magnetic resonance images, the recent advancements in processing power have allowed for a wide variety of fully 3D models to be proposed, offering markedly better performance with respect to the more traditional methods [10,11]. In our study, we compare our proposed method against a series of representative deep architectures, with applications ranging from semantic segmentation to point-cloud classification and medical image segmentation. In particular, we consider the *SegNet* [12], *DenseVoxNet* [13], *VoxResNet* [14], *PointNet* [15], *CAN3D* [13], and *KCB-Net* [16] architectures (Section 9.4.2).

### 1.5. Graph Convolutional Neural Networks

Recently, intensive research has been conducted in the field of graph convolutional networks, owing to their efficiency in handling non-Euclidean data [17]. These models can be distinguished into two general categories, namely, the spectral-based [18,19] and the spatial-based methods [20,21,22,23,24]. The spectral-based networks rely on the graph signal processing principles, utilizing filters to define the node convolutions. The *ChebNet* in [18] approximates the convolutional filters by Chebyshev polynomials of the diagonal matrix of eigenvalues while the *GCN* model in [19] performs a first-order approximation of *ChebNet*.

Spatial-based graph convolutions, on the other hand, update a central node’s representation by aggregating the representations of its neighboring nodes. The message-passing neural network (*MPNN*) [20] considers graph convolutions as a message-passing process, whereby information is traversed between nodes via the graph edges. *GraphSAGE* [25] applies node sampling to obtain a fixed number of neighbors for each node’s aggregation. A graph attention network (*GAT*) [24] assumes that the contribution of the neighboring nodes to the central one is determined according to a relative weight of importance, a task achieved via a shared attention mechanism across nodes with learnable parameters.

During the last years, *GCN*s have found extensive use in a diverse range of applications, including citation and social networks [19,26], graph-to-sequence learning tasks in natural language processing [27], molecular/compound graphs [28], and action recognition [29]. Considerable research has been conducted on the classification of remotely sensed hyperspectral images [30,31,32], mainly due to the capabilities of *GCN*s to capture both the spatial contextual information of pixels, as well as the long-range relationships of distant pixels in the image. Another domain of application is the forecasting of traffic features in smart transportation networks [33,34]. To capture the varying spatio-temporal relationships between nodes, integrated models are developed in these works, which combine graph-based spatial convolutions with temporal convolutions.

Finally, to confront the gradient vanishing effect faced by traditional graph-based models, deep *GCN* networks have recently been suggested [35,36]. Particularly, in [35], a densely connected graph convolutional network (*DCGCN)* is proposed for graph-to-sequence learning, which can capture both local and nonlocal features. In addition, Ref. [36] presents a densely connected block of *GCN* layers, which is used to generate effective shape descriptors from 3D meshes of images.

### 1.6. Outline of Proposed Method

The existing patch-based methods exhibit several drawbacks which can potentially degrade their segmentation performance. First, for each target voxel, these methods construct a local patch library at a specific spatial scale, comprising neighboring voxels from atlas images. Then, classifiers are developed by considering pairwise similarities between voxels in that local region. This suggests that target labeling is accomplished by relying solely on local learning while disregarding the global contextual information among pixels. Hence, long-range relationships among distant voxels in the region of interest are ignored, although these voxels may belong to the same class but with a different textural appearance. Secondly, previous methods in the field resort to inductive learning to produce voxel segmentation, which implies that the features of the unlabeled target voxels are not leveraged during the labeling process. Finally, some recent segmentation methods employ graph-based approaches allowing a more effective description of voxel pairwise affinities via sparse code reconstructions [8,37]. Despite the better data representation, the target voxel labeling is achieved using linear aggregation rules for transferring the atlas voxel’s labels, such as the traditional label propagation (*LP*) mechanism via the graph edges. Such first order methods may fail to adequately capture the full scope of dependencies among the voxel representations. The labeling of each voxel proceeds by aggregating spectral information strictly from its immediate neighborhood, failing to exploit long-term dependencies with potentially more similar patches in distant regions of the image, thus ultimately yielding suboptimal segmentation results.

**Figure 1:**
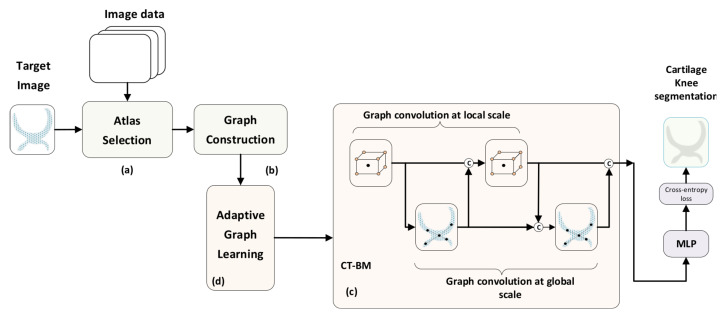
Outline of the proposed knee cartilage segmentation approach. It comprises the atlas subset selection (**a**), the graph construction part (**b**), a specific form of graph-based convolutional model (**c**), the adaptive graph learning (**d**), and the *MLP* network providing the class estimates for the segmentation of the target image. Black dots correspond to central nodes and colored nodes to neighboring ones, respectively. The encircled C symbol represents an aggregation function.

To properly address the above shortcomings, in this paper we present a novel method for the automatic segmentation of knee articular cartilage, based on recent advances in the field of graph-based neural networks. More concretely, we propose the dense multi-scale adaptive graph convolutional network (*DMA-GCN*) method, which constructively integrates local spatial-level learning and global-level contextual learning concurrently. Our goal is to generate, via automatic convolutional learning, expressive node representations by merging pairwise importance at multiple spatial scales with long-range dependencies among nodes for enhanced volume segmentation. We approach the segmentation task as a multi-class classification problem with the five classes: background: 0; femoral bone: 1; femoral cartilage: 2; tibial bone: 3; tibial cartilage: 4. Recognizing the more crucial role of the cartilage structure in the assessment of the knee joint and considering the increased difficulty for its automatic segmentation as contrasted with that of bones, our efforts are primarily devoted to that issue. Figure 1 depicts a schematic framework of the proposed approach. The main properties and innovations of the *DMA-GCN* model are described as follows:

*Multi-atlas setting*: Our scheme is tailored to the multi-atlas approach, whereby label information from atlas images (labeled) is transferred to segment the target image (unlabeled). To this end, at the preliminary stage, images are aligned using a cost-effective affine registration. Subsequently, for each target image T, we generate its corresponding atlas library {A,L} according to a similarity criterion (Figure 1a).

*Graph construction*: This part refers to the way in which images are represented in terms of nodes and the organization of node data to construct the overall graph (Figure 1b). Here, the graph node corresponds to a generic patch of size 5 × 5 × 5 around a central voxel, while the node feature vector is provided by a 3D-HOG feature descriptor. Accordingly, the image is represented as a collection of spatially stratified nodes, covering adequately all classes across the region of interest. Following the multi-atlas setting, we construct sequences of aligned data, comprising target nodes and those for the atlases at spatially correspondent locations. Given the node sequences, we further generate the sequence libraries which are composed of neighboring nodes at various spatial scales. The collection of all node libraries forms the overall graph structure, whereby both local (spatially neighboring) and global (spatially distant) node relationships are incorporated.

*Semi-supervised learning (SSL):* Following the *SSL* scenario, the input graph data comprise both labeled nodes from the atlas library and unlabeled ones from the target image to be segmented. In that respect, contrary to some existing methods, the features of unlabeled data are leveraged via learning to compute the node embbeddings and label the target nodes.

*Local–global learning:* As can be seen from Figure 1c, graph convolutions over the layers proceed along two directions, namely, the local spatial level and the global level, respectively. The local spatial branch includes the so-called local convolutional (Lconv) units which operate on the subgraph of aligned neighborhoods of nodes (sequence libraries). The node embeddings generated by these units incorporate the contextual information between nodes at a local spatial level. To further improve local search, we integrate local convolutions at multiple scales, so that the local context around nodes is captured more efficiently. On the other hand, the global branch includes global convolution (Gconv) units. These units provide the global node embeddings by taking into consideration the pairwise affinities of distant nodes distributed over the entire region of interest of the cartilage volume. The final node representations are then obtained by aggregating the embeddings computed at the local spatial and the global levels, respectively.

*Convolutional building models:* An important issue is how the local and global hidden representations of nodes are combined across the convolution layers. In this context, we propose two different structures: the cross-talk building model (*CT-BM*) and the sequential building model (*SEQ-BM*). Both models comprise four convolutional units overall, specifically, two Lconv and two Gconv units, undertaking local and global convolutions, respectively. The *CT-BM* (Figure 1c) performs intertwined local–global learning, with skip connections and aggregators. The links indicate the cross-talks between the two paths. The *SEQ-BM*, on the other hand, adopts a sequential learning scheme. In particular, local spatial learning is completed first, followed by the respective convolutions at the global level.

*Adaptive graph learning (AGL):* Considering fixed graphs with predetermined adjacency weights among nodes can degrade the segmentation results. To confront this drawback, every Lconv and Gconv unit is equipped here with an *AGL* mechanism, which allows us to automatically learn the proper graph structure at each layer. At the local spatial level, *AGL* adaptively designates the connectivity relationships between nodes via learnable attention coefficients. Hence, Lconv can concentrate and aggregate features from relevant nodes in the local search region. Further, at the global level we propose a different *AGL* scheme for Gconv units, whereby graph edges are learned from the input features of each layer.

*Densely connected GCN:* The proposed *CT-BM* and *SEQ-BM* can be utilized as standalone models to undertake the graph convolution task. Nevertheless, their depth is confined to two local–global layers, since an attempt to deepen the networks is hindered by the gradient-vanishing effect. To circumvent this deficiency, we finally propose a densely connected convolutional network, the *DMA-GCN* model. The *DMA-GCN* considers *CT-BM* or *SEQ-BM* as the building block of the deep structure. It exhibits a deep architecture with skip connections whereby each layer in the block receives feature maps from all previous layers and transmits its outputs to all subsequent layers. Overall, the *DMA-GCN* shares some salient qualities, such as a deep structure with an enhanced performance rate and better information flow, local–global level convolutions, and adaptive graph learning.

In summary, the main contributions of this paper are described as follows.

- A novel multi-atlas approach is presented for knee cartilage segmentation from *MRI* images based on graph convolutional networks which operates under the semi-supervised learning paradigm.
- With the aim to generate expressive node representations, we propose a new learning scheme that integrates graph information at both local and global levels concurrently. The local branch exploits the relevant spatial information of neighboring nodes at multiple scales, while the global branch incorporates global contextual relationships among distant nodes.
- We propose two convolutional building models, the *CT-BM* and *SEQ-BM*. In the *CT-BM*, the local and global learning tasks are intertwined along the layer convolutions, while the *SEQ-BM* follows a sequential mode.
- Both local and global convolutional units, at each layer, are equipped with suitable attention mechanisms, which allows the network to automatically learn the graph connective relationships among nodes during training.
- Using the proposed *CT-BM* and *SEQ-BM* as block units, we finally present a novel densely connected model, the *DMA-GCN*. The network exhibits a deeper structure which leads to more enhanced segmentation results, while at the same time, it shares all salient properties of our approach.
- We have devised a thorough experimental setup to investigate the capabilities of the suggested segmentation framework. In this setting, we examine different test cases and provide an extensive comparative analysis with other segmentation methods.

The remainder of this paper is organized as follows. Section 2 reviews some representative forms of graph convolutional networks related to our work and involved in the experimental analysis. Section 3 presents the image preprocessing steps and the atlas selection process. In Section 4, we discuss the node feature descriptor, as well as the graph construction of the images. Section 5 elaborates on the proposed local and global convolutional units, along with their attention mechanisms. Section 6 describes the suggested convolutional building blocks, while Section 7 presents our densely connected network. Section 8 discusses the transductive vs. inductive learning and the full-batch vs. mini-batch learning in our approach. In Section 9 and Section 10, we provide the experimental setup and respective comparative results of the proposed methodology, while Section 11 concludes this study.

## 2. Related Work

In this section, we review some representative models in the field of graph convolutional networks that are related to our work and are also included in the experiments.

**Definition** **1.** *A graph G is defined as G=(V,E,X) where V denotes the set of N nodes $v_{i}\in V$, and E is the set of edges connecting the nodes $(v_{i},v_{j})\in E$. $X\in R^{N\times F}$ is a matrix subsuming the node feature descriptors $x_{i}\in R^{D},i=1,\ldots N$ with F denoting the feature vector dimensionality. The graph is associated with an adjacency matrix $A\in R^{N\times N}$ (binary or weighted), which includes the connection links between nodes. A larger entry $A_{ij}>0$ suggests the existence of a strong relationship between nodes $\left(v_{i},v_{j}\right)$, while $A_{ij}=0$ signifies the lack of connectivity. The graph Laplacian matrix $L\in R^{\left(N\times N\right)}$ is defined as L=D−A, where D is the diagonal degree matrix with $D_{ii}=\sum_{j=1}^{N}A_{ij},i=1,\ldots ,N$. Finally, the normalized graph adjacency matrix with the added self-connections is denoted by $\tilde{A}=A+I_{N}$, with the corresponding degree matrix given by ${\tilde{D}}_{ii}=\sum_{j=1}^{N}{\tilde{A}}_{ij}$*

### 2.1. Graph Convolutional Network (GCN)

The *GCN* proposed in [19] is a spectral convolutional model. It tackles the node classification task under the semi-supervised framework, i.e., where labels are available only for a portion of the nodes in the graph. Under this setting, learning is achieved by enforcing a graph Laplacian regularization term with the aim of smoothing the node labels:

(1) $$\begin{array}{cc} L & =L_{0}+\lambda L_{reg} \end{array}$$

(2) $$\begin{array}{cc} L_{reg} & =\sum_{i}\sum_{j}A_{ij}∥f\left(x_{i}\right)-f\left(x_{j}\right)∥=f{\left(X\right)}^{T}Lf\left(X\right) \end{array}$$

where $L_{0}$ represents the supervised loss measured on the labeled nodes of the graph, f(X,A) is a differentiable function implemented by a graph neural network, λ is a regularization term balancing the supervised loss in regard to the overall smoothness of the graph, X is the node feature matrix, and L is the graph Laplacian.

In a standard multilayer graph-based neural network framework, information flows across the nodes by applying the following layerwise propagation rule:

(3) $$H^{\left(l+1\right)}=\sigma \left({\tilde{D}}^{-\frac{1}{2}}\tilde{A}{\tilde{D}}^{-\frac{1}{2}}H^{\left(l\right)}W^{\left(l\right)}\right)$$

where σ(·) denotes the LeakyReLU(·) activation function, $W^{\left(l\right)}$ is a layer-specific trainable weight matrix, and $H^{\left(l\right)}$ is the matrix of activation functions in the *l*th layer, with $H^{\left(0\right)}=X$. The authors in [19] show that the propagation rule in Equation (3) provides a first-order approximation of localized spectral filters on graphs. Most importantly, we can construct multilayered graph convolution networks by stacking several convolutional layers of the form in Equation (3). For instance, a two-layered *GCN* can be represented by

(4) $$Z=f\left(X,A\right)=softmax\left(\hat{A}ReLU\left(\hat{A}XW^{\left(0\right)}\right)W^{\left(1\right)}\right)$$

where Z is the network’s output, softmax(·) is the output layer activation function for multi-class problems, and $\hat{A}={\tilde{D}}^{-\frac{1}{2}}\tilde{A}{\tilde{D}}^{-\frac{1}{2}}$ is the normalized adjacency matrix. The weight matrices $W^{\left(0\right)}$ and $W^{\left(1\right)}$ are trained using some variant of gradient descent with the aid of a loss function.

### 2.2. Graph Attention Network (GAT)

A salient component in *GATs* [24] is an attention mechanism incorporated in the aggregation of the graph attention layers (*GALs)*, with the aim to automatically capture valuable relationships between neighboring nodes. Let $H=\left\{h_{1},\ldots ,h_{N}\right\},h_{i}\in R^{D}$ and $\tilde{H}=\left\{{\tilde{h}}_{1},\ldots ,{\tilde{h}}_{N}\right\},{\tilde{h}}_{i}\in R^{\tilde{D}}$ denote the inputs and outputs of a *GAL*, where *N* is the number of nodes, while *D* and $\tilde{D}$ are the corresponding dimensionalities of the node feature vectors. The convolution process entails three distinct issues: the shared node embeddings, the attention mechanism, and the update of node representations. As an initial step, a learnable transformation parameterized by the weight matrix $W\in R^{\tilde{D}\times D}$ is applied on nodes, with the goal of producing expressive feature representations. Next, for every node pair, a shared attention mechanism is performed on the transformed features,

(5) $$g_{ij}=\alpha^{T}\cdot \left(Wh_{i}\left|\right|Wh_{j}\right)$$

where $g_{ij}$ signifies the importance between nodes $h_{i}$ and $h_{j}$, α is a learnable weight vector, and || denotes the concatenation operator. To make the above mechanism effective, the computation of the attention coefficients is confined between each node −i and its neighboring nodes −j, $j\in N_{i}$. In the *GAT* framework, the attention mechanism is implemented by a single-layer feed-forward neural network, parameterized by LeakyReLU nonlinearities, which provide the normalized attention coefficients:

(6) $$a_{ij}=\frac{\exp\left(LeakyReLU\left(\alpha^{T}\cdot [Wh_{i}||Wh_{j}]\right)\right)}{\sum_{k\in N_{i}}\exp\left(LeakyReLU\left(\alpha^{T}\cdot [Wh_{i}||Wh_{k}]\right)\right)}$$

Given the attention coefficients, the node feature representations at the output of the *GAT* are updated via a linear aggregation of neighboring nodes’ features

(7) $${\tilde{h}}_{i}=\sigma \left(\sum_{j\in N_{i}}\alpha_{ij}Wh_{j}\right)$$

To stabilize the learning procedure, the previous approach is extended in a *GAT* by considering multiple attention heads. In that case, the node features are computed by

(8) $${\tilde{h}}_{i}=\overset{K}{\underset{k=1}{\left|\right|}}\sigma \left(\sum_{j\in N_{i}}\alpha_{ij}^{\left(k\right)}W^{\left(k\right)}h_{j}\right)$$

where *K* is the number of independent attention heads applied, while $\alpha_{ij}^{\left(k\right)}$ and $W^{\left(k\right)}$ denote the normalized attention coefficients associated with the *k*th attention head and its corresponding embedding matrix, respectively. In this work, we exploit the principles of the *GAT*-based attention mechanism in the proposed local convolutional units, with the goal to aggregate valuable contextual information from local neighborhoods, at multiple search scales (Section 5.1).

### 2.3. GraphSAGE

The *GraphSAGE* network in [25] tackles the inductive learning problem, where labels must be generated for previously unseen nodes, or even entirely new subgraphs. *GraphSAGE* aims to learn a set of aggregator functions $AGGR_{k},k=1,\ldots ,K$, which are used to aggregate information from each node’s local neighborhood. Node aggregation is carried out at multiple spatial scales (hops). Among the different schemes proposed in [25], in our experiments, we consider the max-pooling aggregator, where each neighbor’s vector is independently supplied to a fully connected neural network:

(9) $$h_{N_{v}}^{k}=AGGR_{k}^{pool}=\max\left(\left\{\sigma \left(W_{pool}h_{u_{i}}^{k}+b\right),\forall u_{i}\in N\left(v\right)\right\}\right)$$

where max(·) denotes the element-wise max operator, $W_{pool}$ is the weight matrix of learnable parameters, b is the bias vector, and σ(·) is a nonlinear activation function. Further, $h_{N(v)}^{k}$ denotes the result obtained after a max-pooling aggregation on the neighboring nodes of node *v*. *GraphSAGE* then concatenates the current node’s representation $h_{v}^{k-1}$ with the aggregated neighborhood feature vector $h_{N(v)}^{k-1}$ to compute, via a fully connected layer, the updated node feature representations:

(10) $$h_{v}^{k}=\sigma \left(W^{k}\cdot \left(h_{v}^{k-1}\left|\right|h_{N_{v}}^{k-1}\right)\right)$$

where $W^{k}$ is a weight matrix associated with aggregator $AGGR_{k}^{pool}$.

### 2.4. GraphSAINT

*GraphSAINT* [21] differs from the previously examined architectures in that instead of building a full *GCN* on all the available training data, it samples the training graph itself, creating subsets of the original graph, building and training the associated *GCN*s on those subgraphs. For each mini-batch sampled in this iterative process, a subgraph $G_{s}=\left(V_{s},E_{s}\right)$ (where $|V_{s}\left|\ll |V\right|$) is used to construct a GCN. Forward and backward propagation is performed, updating the node representations and the participating edge weights. An initial preprocessing step is required for the smooth operation of the process, whereby an appropriate probability of sampling must be assigned to each node and edge of the initial graph.

## 3. Materials

In this section, we present the dataset used in this study, the image preprocessing steps, and finally, the construction of the atlas library.

### 3.1. Image Dataset

**Figure 2:**
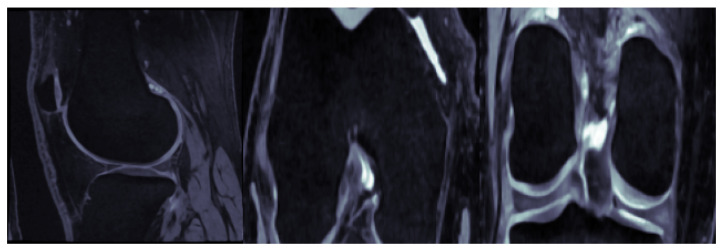
A typical knee MRI viewed in three orthogonal planes (**left** to **right**: sagittal, coronal, axial).

The MR images used in this study comprise the entirety of the publicly available, baseline Osteoarthritis Initiative (*OAI*) repository, for which segmentation masks are available, consisting of a total of 507 subjects. The specific MRI modality utilized across all the experiments corresponds to the sagittal 3D dual-echo steady-state (*3D-DESS*) sequence with water excitation, with an image size of 384×384×384 voxels and a voxel size of 0.36×0.36×0.70 mm. The respective segmentation masks serving as the ground truth are provided by the publicly available repository assembled by [38], including labels for the following knee joint structures (classes): background tissue, femoral bone (*FB*), femoral cartilage (*FC*), tibial bone (*TB*), and tibial cartilage (*TC*). Figure 2 showcases a typical knee MRI, in the three standard orthogonal planes (sagittal, coronal, axial).

### 3.2. Image Preprocessing

The primary source of difficulties in automated cartilage segmentation stems from the similar texture and intensity profile of articular cartilage and background tissues, as they are depicted in most MRI modalities, a problem further accentuated by the usually high intersubject variability present in the imaging data. To this end, the images were preprocessed by applying the following steps:

1. *Curvature flow filtering:* A denoising curvature flow filter [39] was applied, with the aim of smoothing the homogeneous image regions, while simultaneously leaving the surface boundaries intact.
2. *Inhomogeneity correction: N3* intensity nonuniform bias field correction [40] was performed on all images, dealing with the issue of intrasubject variability within similar classes among subjects.
3. *Intensity standardization:* MRI histograms were mapped to a common template, as described in [41], ensuring that all associated structures across the subjects shared a similar intensity profile.
4. *Nonlocal-means filtering:* A final filtering process smoothed out any leftover artefacts and further reduced noise. The method presented in [42] offers a robust performance and is widely employed in similar medical imaging applications. Finally, the intensity range of all images was rescaled to [0,100].

### 3.3. Atlas Selection and ROI Extraction

The construction of an atlas library for each target image T to be segmented necessitates the registration of all atlases ${\left\{A_{i}\right\}}_{i=1}^{n_{A}}$ to the particular target image. An affine transformation was employed, registering all atlas images in the target image domain space, accounting for deformations of linear nature, such as rotations, translations, shearing, scaling, etc. The same transformation was also applied to the corresponding label map $L_{i}$ of each atlas, resulting in the atlas library ${\left\{A_{i}^{T},L_{i}^{T}\right\}}_{i=1}^{N_{A}}$ registered to T.

Considering the fact that the cartilage volume accounts for a very small percentage of the overall image volume, a region of interest (*ROI)* was defined for every target image, covering the entire cartilage structure and its surrounding area. A presegmentation mask was constructed by passing the registered atlas cartilage mask through a majority voting (*MV*) filter, and then expanded by a binary morphological dilation filter, yielding the *ROI* for the target image. This region corresponded to the sampling volume for the target image T and its corresponding atlas library ${\left\{A_{i},L_{i}\right\}}_{i=1}^{N_{A}}$. This process guaranteed that the selected *ROI* enclosed the totality of cartilage tissue both in the target T, as well as in the corresponding atlas library.

Finally, to simultaneously reduce the computational load and increase the spatial correspondence between target and atlas images, we included a final atlas selection step. Measuring the spatial misalignment in the *ROI* of every pair ${\left\{T,A_{i}\right\}}_{i=1}^{T}$ using the mean squared difference $\left(MSD_{i}^{ROI}\right)$, we only kept the first $N_{A}$ atlases exhibiting the least disagreement in the metric [37].

## 4. Graph Constructions

In this section, we describe the node representation, the construction of aligned sequences of nodes, and the sequence libraries, which lead to the formation of the aligned image graphs used in the convolutions.

### 4.1. Node Representation

An important issue to properly address is how an image is transformed to a graph structure of nodes. In our setting, a node was described by a generic 5×5×5 patch $p_{i}=p\left(x_{i}\right)$ surrounding a central voxel $x_{i}$. The image was then represented by a collection of nodes which were spatially distributed across the *ROI* volume.

Each node was described by a feature vector $x_{i}=f_{enc}\left(p_{i}\right)\in R^{20}$ implemented via *HOG* descriptors [43], which aggregated the local information on the node patch. *HOG* descriptors constitute a staple feature descriptor in image processing and recognition. Here, we applied a modification suitable for operating on 3D data [44]. For each voxel $x_{i}$, we extracted an *HOG* feature description by computing the gradient magnitude and direction along the x−y−z axes for each constituent voxel in the node patch. The resulting values were binned to a (q=20)-dimensional feature vector where each entry corresponded to the vertex of a regular icosahedron, with each bin representing the strength of the gradient along that particular direction.

Finally, each node was associated with a class indicator vector $y_{i}=\left[y_{i_{1}},\ldots ,y_{i,c}\right]\in R^{c}$, where $y_{i,c}=1$ if voxel $x_{i}$ belongs in class *i* and 0 otherwise.

### 4.2. Aligned Image Graphs

The underlying principle of the multi-atlas approach is that the target image T and the atlas library $A_{i}^{T}$ are aligned via affine registration, thus sharing a common coordinate space. This allows the transfer of label information from atlas images towards the target one by operating upon sequences of spatially correspondent voxels. Complying with the multi-atlas setting, we applied a two-stage sampling process with the aim to construct a sequence of aligned graphs, involving the target image and its respective atlases. This sequence contained the so-called root nodes which were distinguished from the neighboring nodes introduced in the sequel.

**Figure 3:**
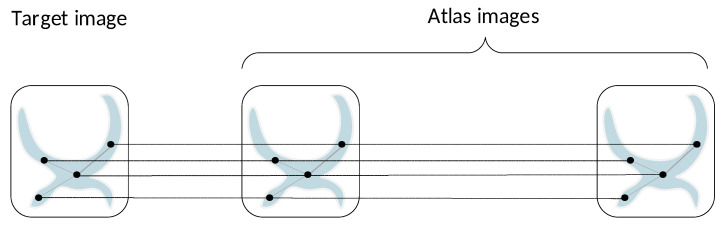
Schematic illustration of a sequence of aligned image graphs of root nodes, including the target graph (**left**) and the graphs of its corresponding atlases (**right**). There are local spatial affinities at aligned positions (horizontal axis), as well as global pairwise similarities between nodes located at different positions in the *ROI*s.

1. *Target graph construction*: This step used a spatially stratified sampling method to generate an initial set of target voxels $X_{r}^{T}\in R^{n_{T}\times D}$, where *D* denotes the feature dimensionality. To ensure a uniform spatial covering of all classes in the target *ROI*, we performed a spatial clustering step partitioning all contained voxels into $n_{r}^{T}$ clusters. After interpolating the cluster centers to the nearest grid point, we obtained the global dataset $X_{r}^{T}=\left\{x_{r}^{T}\left(i\right),i=1,\ldots ,n_{r}^{T}\right\}$, which defined a corresponding target graph of root nodes $G_{r}^{T}$. These target nodes served as reference points from which the aligned sequences were subsequently generated.
2. *Sequences of aligned data*: For each $x_{r}^{T}\left(i\right)\in X_{r}^{T}$, we defined a sequence of aligned nodes S(i), containing the target node $x_{r}^{T}\left(i\right)$ and its respective nodes from the atlas library, located at spatially correspondent positions:
  (11) $$S\left(i\right)=\left\{x_{r}^{T}\left(i\right);x_{r}^{A_{1}}\left(i\right),\ldots ,x_{r}^{A_{n_{A}}}\left(i\right)\right\},\;\forall i=1,\ldots n_{T}$$
  The entire global dataset of root nodes, containing all sampled target nodes and their associated atlas ones, was defined as the union of all those sequences
  (12) $$X_{r}=\overset{n_{T}}{\underset{i=1}{⋃}}S(i)=\left[X_{r}^{T},X_{r}^{A}\right]$$
  where $X_{r}\in R\left(N_{r}\times D\right)$ contains a total number of $N_{r}=n_{T}\cdot \left(n_{A+1}\right)$ root nodes, while $X_{r}^{T}$ and $X_{r}^{A}$ denote the datasets of root nodes sampled from the target and atlases, respectively. Accordingly, this led to a sequence of aligned graphs $G_{r}=\{G_{r}^{T};G_{r}^{A_{1}}$, $\ldots G_{r}^{A_{n_{A}}}\}$, which is schematically shown in Figure 3. In this figure, we can distinguish two modes of pairwise relationships among root nodes that should be explored. Concretely, there are local spatial affinities across the horizontal axis between nodes belonging to a specific node sequence. On the other hand, there also exist global pairwise affinities between nodes of each image individually, as well as between nodes belonging to different images in the sequence. The latter type of search ensures that nodes of the same class located at different positions in the ROI volume and with different textural appearance are taken into consideration, thus leading to the extraction of more expressive node representations of the classes via learning.

### 4.3. Sequence Libraries

**Figure 4:**
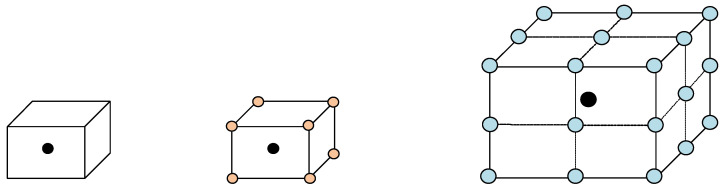
A generic 5×5×5 patch (**left**) representing a node (s=0). The corresponding 1-hop (s=1, **middle**) and 2-hop neighborhoods (s=2, **right**), corresponding to 9×9×9 and 13×13×13 hypercubes, respectively. Black dots correspond to root nodes, while colored ones stand for the neighboring nodes. All nodes are represented by 5×5×5 patches.

It should be stressed that the cost-effective affine registration used in our method is not capable of coping with severe image deformations. Hence, it cannot provide sufficiently accurate alignment between the target and the atlases. To account for this deficiency, we expanded the domain of local search by considering neighborhoods around nodes. Specifically, for each node $x_{i}$, we defined multihop neighborhoods at multiple scales:

(13) $$R_{s}\left(x_{i}\right)=R_{s-1}\left(x_{i}\right)\cup R_{1}\left(R_{s-1}\left(x_{i}\right)\right)$$

for s=1,…,S, where $R_{s}\left(x_{i}\right)$ denotes the neighborhood at scale *s*, and *S* is the number of scales used. $R_{0}\left(x_{i}\right)=x_{i}$ corresponds to the basic patch 5×5×5 of the node itself. $R_{1}\left(x_{i}\right)$ and $R_{2}\left(x_{i}\right)$ are the 1-hop and 2-hop neighborhoods delineated as 9×9×9 and 13×13×13 volumes around $x_{i}$, respectively. In our experiments, we considered two different spatial scales (S=2). Figure 4 illustrates the different node neighborhoods.

Next, for each sequence $S\left(i\right)\;i=1,\cdots ,n_{T}$, we created the corresponding sequence libraries by incorporating the local neighborhoods of all root nodes belonging to that sequence. The sequence library ${\mathrm{SL}}_{s}\left(i\right)$ at scales s=0,1,⋯,S was defined by

(14) $${\mathrm{SL}}_{s}\left(i\right)=R_{s}\left(x_{r}^{T}\left(i\right)\right)\cup \left\{R_{s}\left(x_{r}^{A_{1}}\left(i\right)\right)\cup \ldots \cup R_{s}\left(x_{r}^{A_{n_{A}}}\left(i\right)\right)\right\}$$

**Figure 5:**
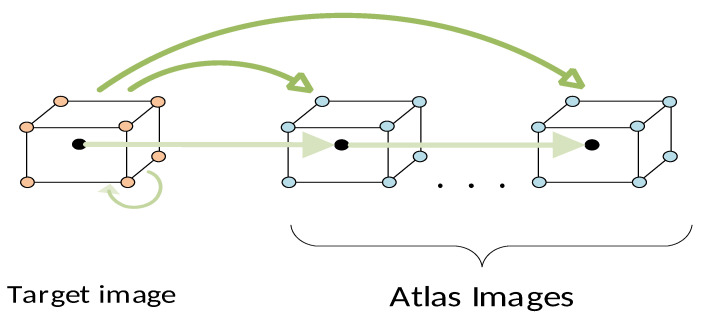
Schematic illustration of a sequence library for a specific scale s=1, comprising the aligned neighborhoods from the target and the atlas images. Green arrows indicate the different scopes of the attention mechanism. For a particular root node, attention is paid to its own neighborhood, as well as the other neighborhoods in the sequence.

${\mathrm{SL}}_{s}\left(i\right)$ contains $\left(n_{A}+1\right)\cdot \left|R_{s}\right|$ nodes, where $|R_{s}|$ denotes the size of the spatial neighborhood at scale *s*. Figure 5 provides a schematic illustration of a sequence library.

The collection of all ${\mathrm{SL}}_{s}$ forms a global dataset $X_{s}$ of aligned neighborhoods described as follows:

(15) $$X_{s}=\overset{n_{T}}{\underset{i=1}{⋃}}{\mathrm{SL}}_{s}\left(i\right)=X_{r}\cup N_{s}$$

for s=1,…,S. $X_{s}$ is formed as union of the dataset $X_{r}$ of root nodes and the dataset $N_{s}$ comprising their neighboring nodes at scale *s*. Its cardinality is $|X_{s}|=N_{r}+\left|N_{s}\right|$, where $N_{r}$ is the number of root nodes, and $|N_{s}|$ the cardinality of $N_{s}$. Further, $X_{s}$ corresponds to a subgraph $G_{s}\left(V_{s},E_{s}\right)$, with $|V_{s}\left|=\right|X_{s}|$. In this subgraph, connective edges are established in $E_{s}$ along the horizontal axis, namely, between root nodes and the neighboring nodes across the sequence libraries. Concluding, since the neighborhoods are by definition inclusive as the scale increases, the dataset $X_{S}$ contains the maximum number of nodes, forming the overall dataset $\tilde{X}$:

(16) $$\tilde{X}=X_{S}=\overset{n_{T}}{\underset{i=1}{⋃}}{\mathrm{SL}}_{S}\left(i\right)$$

The corresponding graph G comprises an overall total number of $N=|N_{r}\left|+\right|N_{S}|$ nodes, including the root and neighboring nodes.

## 5. Convolutional Units

**Figure 6:**
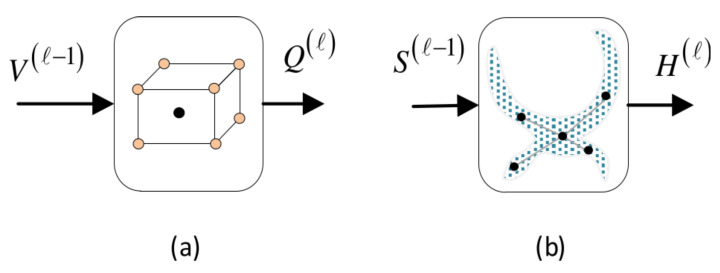
Outline of the convolutional units employed. (**a**) The local convolutional unit (Lconv), (**b**) the global convolutional unit (Gconv).

This section elaborates on the basic convolutional units, namely, the local convolutional unit Lconv and the global convolutional unit Gconv which serve as structural elements to devise our proposed models (Figure 6).

### 5.1. Local Convolutional Unit

The local convolutional unit undertakes the local spatial learning task, operating horizontally along the sequence libraries (*SL_s_)* of nodes. Instead of confining ourselves to predefined and fixed weights in the graphs, we opted to apply a local attention mechanism to adaptively learn the graph structure information at each layer. Specifically, we used the attention approach suggested in the *GAT* as a means to capture the local contextual relationships among nodes in the search area.

**Figure 7:**
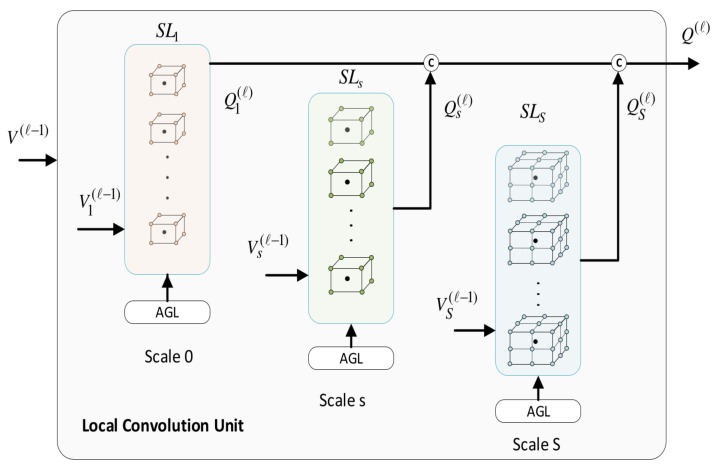
Detailed description of the local convolutional unit, which aggregates local contextual information from node neighborhoods at different spatial scales.

A functional outline of Lconv at layer *l* is shown in Figure 7. It receives an input $V^{\left(l-1\right)}\in R^{N\times E_{in}^{\left(l-1\right)}}$ from the previous layer and provides its output $Q^{\left(l\right)}\in R^{N\times E_{o}^{\left(l\right)}}$, where $E_{in}^{\left(l-1\right)}$ and $E_{o}^{\left(l\right)}$ denote the dimensionalities of the input and output node features, respectively.

Figure 7 provides a detailed architecture of Lconv. The model involves *S* sub-modules, each one associated with a specific spatial scale of aggregation. The sub-module *s* acts upon the subgraph $G_{s}\left(V_{s},E_{s}\right),s=0,\ldots ,S$, which subsumes the sequence libraries ${\mathrm{SL}}_{s}$ of root nodes. Its input is $V_{s}\in R^{|X_{s}|\times E_{in}^{\left(l-1\right)}}$ and after a local convolution at scale s, it provides its own output $Q_{s}^{\left(l\right)}\in R^{|X_{s}|\times E_{o}^{\left(l\right)}}$. In this context, the structure of $G_{s}$ is adapted to the local attention mechanism. Let us assume that a root node $x_{i}$ belongs to the *q*th sequence library: $x_{i}\in {\mathrm{SL}}_{s}\left(q\right),\;q=1,\ldots ,n_{A}$. Then, node $x_{i}$ pays attention to two pools of neighboring nodes (Figure 3): (a) it aggregates relevant feature information from nodes $x_{j}$ of its own neighborhood, $x_{j}\in R_{s}\left(x_{i}\right)$ (self-neighborhood attention); (b) it aggregates features of nodes belonging to the other aligned neighborhoods in ${\mathrm{SL}}_{s}\left(q\right)$ pertaining to the atlas images: $x_{j}\in {\mathrm{SL}}_{s}\left(q\right)\setminus R_{s}\left(x_{i}\right)$. For these pairs of nodes, we compute normalized attention coefficients using Equation (6). Further, pairwise affinities between nodes belonging to different sequence libraries are disregarded, i.e., $\alpha_{ij}=0$ when $x_{i}\in {\mathrm{SL}}_{s}\left(q\right)$ and $x_{j}\in {\mathrm{SL}}_{s}\left(p\right)$, p≠q. It should be noticed that we are primarily focused on computing comprehensive feature representations of the root nodes. Nevertheless, neighboring nodes are also updated; however, in this case, the attention is confined to the neighborhood of the root node it belongs to.

For convenience, let us consider the input node features of the form $V_{s}^{\left(l-1\right)}=\left[v_{s,1}^{\left(l-1\right)},\ldots ,v_{s,|X_{s}|}^{\left(l-1\right)}\right]$, where $v_{s,i}\in R^{E_{in}^{\left(l-1\right)}}$ and similarly, $Q_{s}^{\left(l\right)}\in \left[q_{s,1}^{\left(l\right)},\ldots ,q_{s,|X_{s}|}^{\left(l\right)}\right],q_{s,i}^{\left(l\right)}\in R^{E_{o}^{\left(l-1\right)}}$. The local-level convolution at scale *s* of a root node $x_{i}\in {\mathrm{SL}}_{s}\left(q\right),\;i=1,\ldots ,N_{r}$ is obtained by:

(17) $$\begin{array}{c} \begin{array}{c} q_{s,i}^{\left(l\right)}=\sigma \left(\sum_{j\in R_{s}\left(x_{i}\right)}\alpha_{ij}^{\left(k\right)}W_{s,k}^{\left(l\right)}v_{s,i}^{\left(l-1\right)}+\sum_{j\in {\mathrm{SL}}_{s}\left(q\right)\setminus R_{s}}\alpha_{ij}^{\left(k\right)}W_{s,k}^{\left(l\right)}v_{s,i}^{\left(l-1\right)}\right) \end{array} \end{array}$$

The first term in the above equation refers to the self-neighborhood attention, which aggregates node features from $R_{s}\left(x_{i}\right)$ within the same image. Moreover, the second term aggregates node information from the other aligned neighborhoods in the sequence. In an attempt to stabilize the learning process and further enhance the local feature representations, we followed a multi-head approach, whereby *K* independent attention mechanisms are applied. Accordingly, the node convolutions proceed as follows:

(18) $$\begin{array}{c} \begin{array}{c} q_{s,i}^{\left(l\right)}=\overset{K}{\underset{k=1}{\left|\right|}}\sigma \left(\sum_{j\in R_{s}\left(x_{i}\right)}\alpha_{ij}^{\left(k\right)}W_{s,k}^{\left(l\right)}v_{s,i}^{\left(l-1\right)}+\sum_{j\in {\mathrm{SL}}_{s}\left(q\right)\setminus R_{s}}\alpha_{ij}^{\left(k\right)}W_{s,k}^{\left(l\right)}v_{s,i}^{\left(l-1\right)}\right) \end{array} \end{array}$$

where $\alpha_{ij}^{\left(k\right)}$ denote the attention coefficients between nodes $x_{i}$ and $x_{j}$ according to the *k*th attention head, while $W_{s,k}^{\left(l\right)}\in R^{E_{in}^{\left(l\right)}\times E_{o}^{\left(l-1\right)}}$ are the corresponding parameter weights used for node embeddings. The attention parameters are shared across all nodes in $G_{s}\left(V_{s},E_{s}\right)$ and are simultaneously learned at each layer *l* and for each spatial scale, individually. The outputs of the different sub-modules are finally aggregated to yield the overall output of the Lconv unit:

(19) $$Q^{\left(l\right)}=Q_{1}^{\left(l\right)}⊕\cdots ⊕Q_{S}^{\left(l\right)}$$

where ⊕ denotes the concatenation operator.

The multilevel attention-based aggregation of valuable contextual information from sequence libraries offers some noticeable assets to our approach: (a) it acquires comprehensive node representations which assist in producing better segmentation results, (b) the graph learning circumvents the inaccuracies caused by affine registration in severe image deformations which may lead to node misclassification.

### 5.2. Global Convolutional Unit

The global convolutional unit conducts the global convolution task; it acts upon the subgraph $G_{r}\left(V_{r},E_{r}\right)$ which includes the sequence of root nodes S(i). Gconv aims at exploring the global contextual relationships among nodes located at different positions in the target image and the atlases (Figure 3). Accordingly, we established in $E_{r}$ suitable pairwise connective weights according to spectral similarity ${\tilde{A}}_{ij}\neq 0$ for nodes $x_{i}\in S\left(p\right)$, $x_{j}\in S\left(q\right),p\neq q$. Node pairs belonging to the same sequence are processed by Lconv units; hence, they are disregarded in this case.

The global convolution at layer *l* is acquired using the spectral convolutional principles in *GCN*:

(20) $$H^{\left(l\right)}=\sigma \left({\tilde{A}}^{\left(l\right)}S^{\left(l-1\right)}W_{g}^{\left(l\right)}\right)$$

where $S^{\left(l-1\right)}\in R^{\left(N\times F_{in}^{\left(l\right)}\right)}$ and $H^{\left(l-1\right)}\in R^{\left(N\times F_{o}^{\left(l\right)}\right)}$ denote the input and output of Gconv, respectively, whereas $F_{in}^{\left(l-1\right)},F_{o}^{\left(l\right)}$ are the corresponding dimensionalities. $W_{g}^{\left(l\right)}$ is the learnable embedding matrix and ${\tilde{A}}^{\left(l\right)}$ is the adjacency matrix, as defined in Section 2.1.

Similar to Lconv, we also incorporated the *AGL* mechanism to Gconv, so that global affinities could be automatically captured at each layer via learning. More concretely, we applied an adaptive scheme whereby the connective weights between nodes are determined from the module’s input signals [45]. The adjacency matrix elements were computed by:

(21) $$\tilde{A}=\sigma \left[\left({\tilde{H}}^{\left(l-1\right)}W_{\phi }\right){\left({\tilde{H}}^{\left(l-1\right)}W_{\phi }\right)}^{T}\right]+I_{N\times N}$$

where ${\tilde{S}}^{\left(l-1\right)}=\mathrm{BN}\left(S^{\left(l-1\right)}\right)$ is obtained after applying batch-normalization to the inputs, σ(·) is the sigmoidal activation function applied on an element-wise operation, and $W_{\phi }$ is the embedding matrix to be learned, shared across all nodes of $G_{r}\left(V_{r},E_{r}\right)$. The adaptation scheme in Equation (21) assigns greater edge values between nodes with high spectral similarity and vice-versa.

In the descriptions above, we considered the *GCN* model equipped with *AGL* as a baseline scheme. Nevertheless, in our experimental investigation, we examined several scenarios whereby the global convolution task was tackled using alternative convolutional models, including *GraphSage*, *GAT*, *GraphSAINT*, etc.

## 6. Proposed Convolutional Building Blocks

In this section, we present two alternative building models, namely, the cross-talk building model (*CT-BM)* and the sequential building model (*SEQ-BM)*. They are distinguished according to the way the local and global convolutional units are blended across the layers. Every constituent local and global unit within the structures has its individual embedding matrix of learnable parameters. Further, it is also equipped with its own *AGL* mechanism for adaptive learning of the graphs, as described in the previous section.

### 6.1. Cross-Talk Building Model (CT-BM)

**Figure 8:**
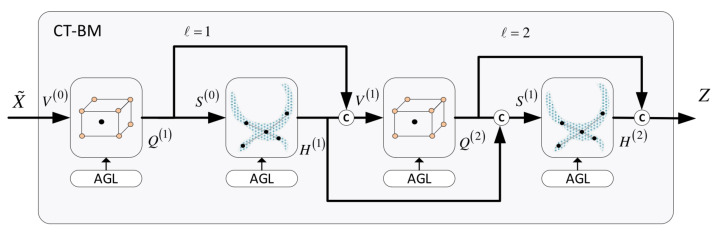
Illustration of the proposed cross-talk building model (*CT-BM)*, where local and global convolutional units are combined following an alternating scheme.

The *CT-BM* is shown in the outline of our approach in Figure 1. Nevertheless, a more compact form is depicted in Figure 8. The model comprises two composite layers (l=1,2), each one containing one local and one global unit. As can be seen, convolutions proceed in an alternating manner across the layers, whereby the local unit transmits its output to the next global unit, and vice versa. A distinguishing feature of this structure is that there are also skip connections and aggregators which implement cross-talk links between the local and global components. Particularly, in addition to the standard flow from one unit to the next, each unit’s output is aggregated with the output of the subsequent unit.

The overall workflow of the *CT-BM* is outlined below:

1. The first local unit yields
  (22) $$V^{\left(0\right)}=X,Q^{\left(1\right)}=Lconv\left(V^{\left(0\right)}\right)$$
2. The local unit’s output is passed to the first global unit to compute
  (23) $$S^{\left(0\right)}=Q^{\left(1\right)},H^{\left(1\right)}=Gconv\left(S^{\left(0\right)}\right)$$
3. The second local unit receives an aggregated signal to provide its output,
  (24) $$V^{\left(1\right)}=H^{\left(1\right)}+Q^{\left(1\right)},\;Q^{\left(2\right)}=Lconv\left(V^{\left(1\right)}\right)$$
4. The second global unit produces
  (25) $$S^{\left(1\right)}=H^{\left(1\right)}+Q^{\left(2\right)},\;H^{\left(2\right)}=Gconv\left(S^{\left(1\right)}\right)$$
5. The final output of the CT-BM is the obtained by
  (26) $$Z=H^{\left(2\right)}+Q^{\left(2\right)}$$

### 6.2. Sequential Building Model (SEQ-BM)

**Figure 9:**
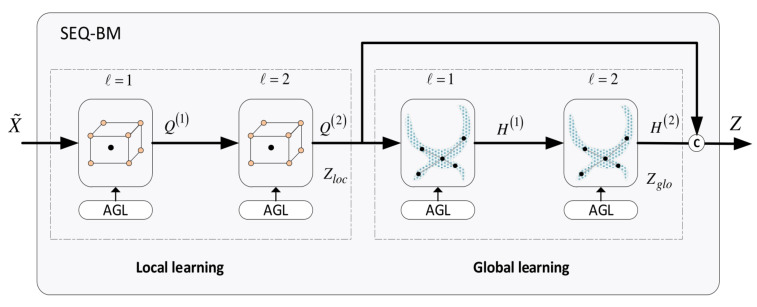
Illustration of the proposed sequential building model (*SEQ-BM)*, where the local and global learning tasks are carried out sequentially.

The architecture of the *SEQ-BM* is illustrated in Figure 9. This model also contains two local and two global units. Contrary to the *CT-BM*, convolutions are conducted in the *SEQ-BM* sequentially. Concretely, the local learning task is first completed using the first two local convolutional units. The outputs of this stage are then transmitted to the subsequent stage which accomplishes the global learning task, using the two global convolutional units. The overall output of the *SEQ-BM* is formed by aggregating the resulting local and global features of the two stages.

The workflow of the *SEQ-BM* is outlined as follows:

1. The local learning task is described by
  (27) $$\begin{array}{cc} V^{\left(0\right)} & =X,Q^{\left(1\right)}=Lconv\left(V^{\left(0\right)}\right) \end{array}$$
  (28) $$\begin{array}{cc} V^{\left(1\right)} & =Q^{\left(1\right)},Q^{\left(2\right)}=Lconv\left(V^{\left(1\right)}\right) \end{array}$$
2. The global learning task is described by
  (29) $$\begin{array}{cc} S^{\left(0\right)} & =Q^{\left(2\right)},H^{\left(1\right)}=Gconv\left(S^{\left(0\right)}\right) \end{array}$$
  (30) $$\begin{array}{cc} S^{\left(1\right)} & =H^{\left(1\right)},H^{\left(2\right)}=Gconv\left(S^{\left(1\right)}\right) \end{array}$$
3. The final output of the SEQ-BM is obtained by
  (31) $$Z=Q^{\left(2\right)}+H^{\left(2\right)}=Z_{loc}+Z_{glo}$$

The alternating blending of the *CT-BM* provides a more effective integration between local and global features at each layer, individually, as compared to the sequential combination in *SEQ-BM*. This observation is attested experimentally as shown in Section 10.

## 7. Proposed Dense Convolutional Networks

In this section, we present two variants of our main model, the *DMA-GCN* network. The motivation behind this design is based on an attempt to further expand the structures *CT-BM* and *SEQ-BM* by including multiple layers of convolutions, to face the gradient vanishing effect, where the gradients diminish, thus hindering effective learning or even worsening the results. This is the reason why in the above building blocks, we are restricted to two-layered local–global convolutions.

**Figure 10:**
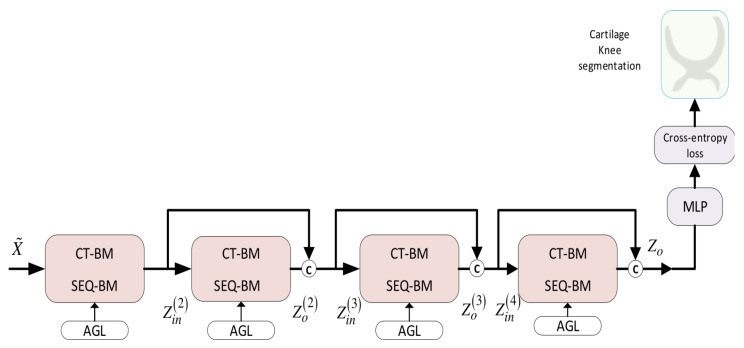
Description of the proposed *DMA-GCN* model, with densely connected block convolutional structure and residual skip connections.

To tackle this problem, we resorted to the recent advancements in deep GCNs [36] and developed the *DMA-GCN* model with a densely connected convolutional architecture utilizing residual skip connections, as shown in Figure 10. As can be seen, the model consists of several blocks arranged across M layers of block convolutions. These blocks are implemented by either *CT-BM* or *SEQ-BM* described in Section 5, which leads to two different alternative configurations, the *DMA-GCN(CT-BM)* and *DMA-GCN(SEQ-BM)*, respectively. Let $Z_{in}^{\left(i\right)}\in R^{\left(N\times P_{in}^{\left(i\right)}\right)}$ and $Z_{o}^{\left(i\right)}\in R^{\left(N\times P_{o}^{\left(i\right)}\right)}$ denote the input and output of the *i*th block, i=1,…,M, with $P_{in}^{\left(i\right)},P_{out}^{\left(i\right)}$ being the feature dimensionalities, respectively. The properties of the suggested *DMA-GCN* are discussed in the following:

1. The skip connections interconnect the blocks across the layers. Concretely, each block receives as input the outputs of blocks from all preceding layers:
  (32) $$Z_{in}^{\left(i\right)}=Z_{o}^{\left(1\right)}⊕Z_{o}^{\left(2\right)}⊕\cdots ⊕Z_{o}^{\left(i-1\right)}$$
  for i=1,…,M, where ⊕ denotes the concatenation operator. This allows the generation of deeper *GCN* structures which can acquire more expressive node features. Overall, the *DMA-GCN* involves 4M convolutional units. Within each block, two layers of local–global convolutions are internally performed; the resulting outputs are then integrated along the block layers to provide the final output:
  (33) $$Z_{o}=Z_{o}^{\left(1\right)}⊕Z_{o}^{\left(2\right)}⊕\ldots ⊕Z_{o}^{\left(M\right)}$$
2. The other beneficial effect of skip connections is that they allow the final output to have direct access to the outputs of all blocks in the dense network. This assures a better reverse flow of information and facilitates the effective learning of parameters pertaining to the blocks. Since block operations are confined to two-layered local–global convolutions, overall, we can circumvent the gradient vanishing problem.
3. In order to preserve the parametric complexity at a reasonable level, similar to [36], we define the feature dimensions of each block in *DMA-GCN* to be the same:
  (34) $$P_{o}^{\left(1\right)}=P_{o}^{\left(2\right)}=\cdots =P_{o}^{\left(M\right)}=d$$
  The node feature growth rate caused by the aggregators can be defined as $P_{in}^{\left(i\right)}=\left(i-1\right)\cdot d$, i=1,⋯,M. The input dimensions grow linearly as we proceed to deeper block layers, with the last block showing the largest increase $P_{in}^{\left(M\right)}=\left(M-1\right)\cdot d$. To prevent feature dimensionalities from receiving too large values, we considered initially a *DMA-GCN* model with M=4 blocks. The particular number of blocks in the above range was then decided after experimental validation (Section 10).
4. Every block in *DMA-GCN* is supported with its corresponding *AGL* process to automatically learn the graph connective affinities at each layer. This is accomplished by applying an attention-based mechanism for local convolutional units (Section 5.1) and an adaptive construction of adjacency matrices from inputs node features (Section 5.2).

The output of the *DMA-GCN* is fed to a two-Layer *MLP* unit to obtain the label estimates

(35) $$\hat{Z_{o}}=\mathrm{softmax}\left(ReLU\left(W_{1}Z\right)W_{2}\right)$$

We adopted the cross-entropy error to penalize the differences between the model’s output ${\hat{Z}}_{o}$ and the corresponding node labels

(36) $$L=-\sum_{l\in Y_{L}}\sum_{c=1}^{C}Y_{lc}Z_{lc}$$

where $Y_{L}$ denotes the subset of labeled nodes. The *DMA-GCN* network was trained under either the transductive or the inductive learning methods, as discussed in the following section.

## 8. Network Learning

In our setting, we considered transductive learning (*SSL*) as the basic learning scheme for training the *DMA-GCN* models. In this case, both unlabeled data from the target image to be segmented T and the labeled data from the atlases LB(T) were used for the construction of the model. Nevertheless, in the experiments, we also investigated the inductive (supervised) learning scenario, whereby training was conducted by solely using labeled data from the atlases.

### 8.1. Transductive Learning (SSL)

The *SSL* scheme was adapted to the context of image segmentation task elaborated here. In regard to the data used in the learning, *SSL* can be carried out along two different modes of operation, namely, mini-batch learning and full-batch learning. Next, we detail mini-batch learning and then conclude with full-batch learning, which is a special case of the former one.

Mini-batch learning was implemented by following a three-stage procedure. In stage 1, an initial model was learned and used to label an initial batch of data from T. Stage 2 was an iterative process, where out-of-sample batches were sequentially sampled from T and labeled via refreshing learning. Finally, stage 3 labeled the remaining voxels of the target image using a majority voting scheme.

*Stage 1: Learning.* In this stage, (t=0), we started by sampling an initial unlabeled batch $X_{r}^{T}\left(0\right)$ from the target image. Then, we used the different steps detailed in Section 4 to construct the corresponding graph of nodes. (a) Given $X_{r}^{T}\left(0\right)$, we created the corresponding aligned sequences (Section 4.3), giving rise to the dataset of root nodes $X_{r}\left(0\right)=\left[X_{r}^{T}\left(0\right),X_{r}^{A}\left(0\right)\right]$. (b) Next, we incorporated neighborhood information by generating sequence libraries at multiple scales (Section 4.3), leading to the datasets $X_{s}\left(0\right)=X_{r}\left(0\right)\cup N_{s}\left(0\right),s=1,\ldots S$, where $N_{s}\left(0\right)$ denotes the neighboring nodes. (c) Finally, we considered the overall dataset $\hat{X}\left(0\right)=X_{S}\left(0\right)$ that corresponded to the graph $G_{0}\left(V_{0},E_{0}\right)$ containing the root and neighboring nodes at that stage.

The next step was to perform convolutional learning on graph $G_{0}\left(V_{0},E_{0}\right)$ using *DMA-GCN* models. Upon completion of the training process, we accomplished the labeling of $X_{r}^{T}\left(0\right)$,

(37) $$l\left(X_{r}^{T}\left(0\right)\right)=F_{\left(0\right)}\left(\tilde{X}\left(0\right),W\left(0\right)\right)$$

$F_{\left(0\right)}$ denotes the model’s functional mapping, l(·) is the labeling function of the target nodes, and W(0) stands for the network’s weights, including the learnable parameters of embedding matrices and attention coefficients, across all layers of the *DMA-GCN*.

*Stage 2: iterative learning.* This stage followed an iterative procedure, t=1,⋯,T, whereby at each iteration, out-of-sample batches of yet unlabeled nodes were sampled from T, $X_{o}^{T}\left(t\right)$ of size $n_{o}$. Considering the nodes in $X_{o}^{T}$ as root nodes, we then applied steps (a)–(c) of the previous stage, to obtain the datasets $X_{o,s}=X_{o}\left(0\right)\cup N_{o,s}\left(0\right),\;s=0,\ldots ,S$, and the overall set ${\tilde{X}}_{o}\left(t\right)=X_{o,S}\left(t\right)$, which corresponded to a graph of out-of-sample nodes. In the following, data ${\tilde{X}}_{o}\left(t\right)$ were fed to the pretrained model from stage 1, $l\left(X_{o}^{T}\right)=F_{\left(t\right)}\left({\tilde{X}}_{o}\left(t\right),W\left(t\right)\right)$. The model was initialized as $W\left(t\right)=W_{0}$ to preserve previously acquired knowledge. Further, it was subject to several epochs of refreshing convolutional learning, with the aim of adapting to the newly presented data. The above sequential process terminated at t=T when all target nodes were labeled.

*Stage 3: labeling of remaining voxels.* This was the final stage of target image segmentation, entailing the labeling of target voxels not considered during the previous learning stages. Given that nodes were the central voxels of a generic 5×5×5 patches, there were multiple remaining voxels scattered within 3×3×3 volumes. Labeling of these voxels was accomplished by a voting scheme. Specifically, for each $x_{r}$, the voting function accounted for both the spectral and the spatial distance from its surrounding labeled vertices:

(38) $$\begin{array}{cc} l(x_{r}) & =\sum_{i}w_{r,i}l\left(x_{i}\right) \end{array}$$

(39) $$\begin{array}{cc} w_{r,i} & =w_{r,i}^{spec}\times w_{r,i}^{spat} \end{array}$$

where $w_{r,i}^{spec}$ and $w_{r,i}^{spat}$ are normalized weighting coefficients denoting the spectral and spatial proximity, measured by the $l_{2}$ norm (Euclidean distance) and $l_{1}$ norm (Manhattan distance), respectively.

Full-batch learning is a special case of the above mini-batch learning. In that case, the dataset $X_{r}^{T}\left(0\right)$ is a large body of data, comprising all possible target nodes contained in the target *ROI*. Under this circumstance, the iterative stage 2 is disregarded. Full-batch learning is completed after convolutional learning (stage 1), followed by the labeling of the rest of target voxels (stage 3).

### 8.2. Inductive Learning

Under this setting, the target data remain unseen during the entire training phase. Adapting to the multi-atlas scenario, we devised a supervised learning scheme according to the following steps. (a) For each target image T, we selected the most similar labeled image $\hat{T}=\mathrm{NN}\left(T\right)$ from atlases, where NN(·) is a spectral similarity function used to identify the nearest neighbors of T. (b) The image $\hat{T}$ along with its corresponding atlas library $\mathrm{LB}(\hat{T})$ were used to learn a supervised model $F_{ind}\left(\hat{T}\right)$ by applying exactly stage 1 of the previous subsection. (c) Finally, the target image T was labeled by means of $F_{ind}\left(\tilde{T}\right)$. As opposed to the *SSL* scenario, the critical difference was that the developed model relied solely on labeled data, disregarding target image information.

## 9. Experimental Setup

### 9.1. Evaluation Metrics

The overall segmentation accuracy achieved by the proposed methods was evaluated using the following three, standard volumetric measures: the Dice similarity coefficient (DSC), the volumetric difference (VD), and the volume overlap error (VOE). Denoting Y the ground truth labels and $\hat{Y}$ the estimated ones, the above measures are defined as:

(40) $$\begin{array}{cc} \mathrm{DSC} & =100\frac{|Y\cap \hat{Y}|}{\left|Y|+\right|\hat{Y}|} \end{array}$$

(41) $$\begin{array}{cc} \mathrm{VOE} & =100\left(1-\frac{\mathrm{DSC}}{200-DSC}\right) \end{array}$$

(42) $$\begin{array}{cc} \mathrm{VD} & =100\frac{|\hat{Y}\left|-|Y\right|}{\left|Y\right|} \end{array}$$

Taking into account that the large majority of voxels correspond to either the background class or the two bone classes, we opted to also include the *precision* and *recall* classification measures, to better evaluate the segmentation performance on each individual structure. All measures correspond exclusively to the image content delineated by the respective *ROI* of each evaluated MRI.

### 9.2. Hyperparameter Setting and Validation

The overall performance of the proposed method depends on a multitude of preset parameters, the most prominent of which are the number of atlases $N_{A}$ comprising the atlas library ${\left\{A,L\right\}}_{i},i=1,\ldots ,N_{A}$, the number of heads *K* utilized in the multi-head attention mechanism, and the number of scales *S* corresponding to the different neighborhood scales. The optimal values of the above hyperparameters, as well as the performance of the *DMA-GCN(SEQ)* and *DMA-GCN(CT)* segmentation methods, were evaluated through a 5-fold cross-validation.

### 9.3. Experimental Test Cases

The proposed methodology comprises several components affecting its overall capacity and performance. We hereby present a series of experimental test cases, aiming to shed light on those effects.

1. *Local vs. global learning*: In this scenario, we aimed to observe the effect of performing local-level learning in addition to global learning. The goal here was to determine the potential boost in performance facilitated by the inclusion of the attention mechanism in our models.
2. *Transductive vs. inductive learning*: The goal here was to ascertain whether the increased cost accompanying the transductive learning scheme could be justified in terms of performance, as compared to the less computationally demanding inductive learning.
3. *Sparse dense adjacency matrix*: Here, we examined the effect of progressively sparsifying the adjacency matrix ${\tilde{A}}^{\left(l\right)}$ at each layer on the overall performance. We examined the following cases: (1) the default case with a dense ${\tilde{A}}^{\left(l\right)}$ and (2) thresholding ${\tilde{A}}^{\left(l\right)}$ so that each node was allowed connections to 5,10, or 20 spectrally adjacent ones.
4. *Global convolution models*: Finally, we tested the effect of varying the design of the global components by examining some prominent architectures, namely, *GCN, ClusterGCN, GraphSAINT, and GraphSAGE*

### 9.4. Competing Cartilage Segmentation Methods

The efficacy of our proposed method was evaluated against several published works dealing with the problem of automatic knee cartilage segmentation.

#### 9.4.1. Patch-Based Methods

The patch-based sparse coding (*PB_SC_*) [8] and patch-based nonlocal-means (*PB_NLM_*) [7] methods are two state-of-the art approaches in medical image segmentation. For consistency reasons, similar to the *DMA-GCN*, we set the patch size for both these methods to (5×5×5) and the corresponding search volume size to (13×13×13). The remaining parameters were taken as described in their respective works.

#### 9.4.2. Deep Learning Methods

Here, we opted to evaluate the *DMA-GCN* against some state-of-the-art deep learning architectures that were successfully applied in the field of medical image segmentation.

*SegNet* [12]: A convolutional encoder–decoder architecture, utilizing the state-of-the-art *VGG16* [46] network that is suitable for pixelwise classification. Since we are dealing with 3D data, we split each input image patch into its constituent planes (−xy,−xz,−yz) and fed those to the network.

*DenseVoxNet* [13]: A convolutional network proposed for cardiovascular MRI segmentation. It comprises a downsampling and upsampling sub-component, utilizing skip connections from each layer to its subsequent ones, enforcing a richer information flow across the layers. In our experiments, the model was trained using the same initialization scheme for the parameters’ values as described in the original paper.

*VoxResNet* [14]: A deep residual network comprising a series of stacked residual modules, each one performing batch-normalization and convolution, also containing skip connections from each module’s input to its respective output.

*KCB-Net* [16]: A recently proposed network that performs cartilage and bone segmentation from volumetric images, by utilizing a modular architecture, where initially, each one of the three sub-components is trained to process a separate plane (sagittal, coronal, axial), followed by a 3D component with the task of aggregating the respective outputs into a single overall segmentation map.

*CAN3D* [47]: This network utilizes a successively dilated convolution kernel aiming to aggregate multi-scale information by performing feature extraction within an increasingly dilating receptive field, facilitating the final voxelwise classification in full resolution. Additionally, the loss function employed at the final layer consists of a combination of Dice similarity coefficient DSC and DSF, a variant of the standard DSC used in evaluating segmentation results.

*Point-Net* [15]: *Point-Net* is a recently proposed architecture specifically geared towards point-cloud classification and segmentation. As an initial preprocessing step, it incorporates a spatial transformer network (*STN*) [48] that renders the input invariant to permutations and is used to produce a global feature for the whole point cloud. That global feature is appended on the output of a standard multilayer perceptron (*MLP*) that operates on the initial point-cloud features, and the resulting aggregated features are passed through another *MLP* in order to provide the final segmentation map.

#### 9.4.3. Graph-Based Deep Learning Methods

In regard to graph-based convolution models, we compared our *DMA-GCN* approach to a series of baseline *GCN* architectures to carry out the global learning task. In addition, we considered in the comparisons the multilevel *GCN* with automatic graph learning (*MGCN-AGL*) method [30], used for the classification of hyperspectral remote sensing images. The *MGCN-AGL* approach takes a form similar to the one of the *SEQ-BM* in Figure 9 to combine the local learning via a *GAT*-based attention mechanism and global learning implemented by a *GCN*. A salient feature of this method is that the global contextual affinities are reconstructed based on the node representations obtained after completion of the local learning stage.

### 9.5. Implementation Details

All models presented in this study were developed using the PyTorch Geometric library (https://github.com/pyg-team/pytorch_geometric, accessed on 1 May 2023), specifically built upon PyTorch (https://pytorch.org) to handle graph neural networks. For the initial registration step, we used the elastix toolkit (https://github.com/SuperElastix/elastix, accessed on 1 May 2023). The code for all models proposed in this study can be found at (https://gitlab.com/christos_chadoulos/graph-neural-networks-for-medical-image-segmentation, accessed on 10 March 2024).

Regarding the network and optimization parameters used in our study, we opted for the following choices: after some initial experimentation, the parameter *d* controlling the node feature growth rate (Section 7) was set to d=128, resulting in the input feature dimensionality progression 128→256→384→512 for M=4 dense layers. All models were trained for 500 epochs using the Adam optimizer, with an early stopping criterion halting the training process either when no further improvement was detected on the validation error in the span of 50 epochs, or when the validation error steadily increased for more than 10 consecutive epochs.

## 10. Experimental Results

### 10.1. Parameter Sensitivity Analysis

In this section, we examine the effect of critical hyperparameters in the performance of the models under examination. The numbers and figures presented for each hyperparameter correspond to results obtained while the remaining ones assumed their optimal determined value.

#### 10.1.1. Number of Selected Atlases $N_{A}$

**Figure 11:**
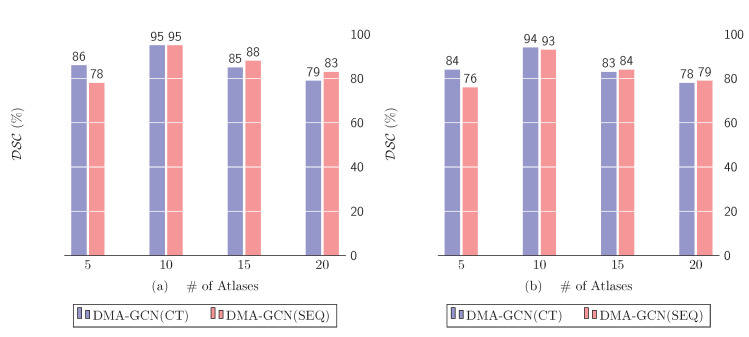
Cartilage DSC(%) score vs. number of atlases. (**a**) Femoral cart, (**b**) tibial cart.

The number of selected atlases is a crucial parameter for all methods adopting the multi-atlas framework. Figure 11 shows the effect on the performance of *DMA-GCN(CT)* and *DMA-GCN(SEQ)* by sampling the following values $N_{A}=5,\ldots ,20$.

For both methods, the number of atlases has a similar effect on the overall performance. The highest score in each case is achieved for $N_{A}=10$ atlases and slowly diminishes as that number grows. Constructing the graph by sampling voxels from a small pool of atlases increases the bias of the model, thus failing to capture the underlying structure of the data. Increasing that number allows the image graphs to include a greater percentage of nodes with dissimilar feature descriptions, which enhances the expressive power of features. Accordingly, a moderate number of aligned atlases seems to provide the best overall rates, as it achieves a reasonable balance between bias and variance.

#### 10.1.2. Number of Attention Heads

**Figure 12:**
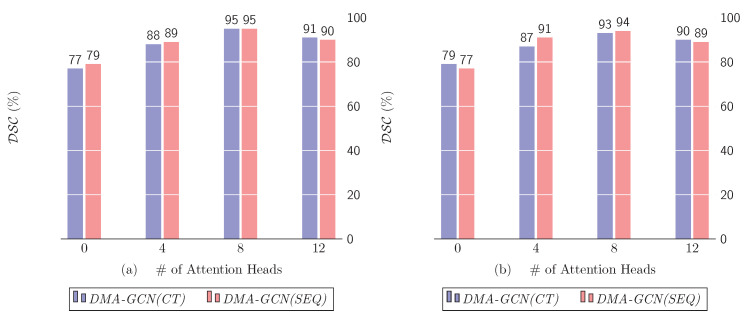
Cartilage DSC(%) score vs. number of attention heads. (**a**) Femoral cart, (**b**) tibial cart.

The number of attention heads is arguably one of the most influential parameters for the local units of our models. Figure 12 demonstrates the effect on performance for K=0,4,8,12. The trivial case of K=0 corresponds to the case where the local convolutional units are disregarded, i.e., the convolution task is undertaken solely by the global units.

The best performance is achieved for K=8 for either *DMA-GCN(CT)* or *DMA-GCN(SEQ)*. Most importantly, in both charts, we can notice a sharp drop in performance when K=0. This indicates that discarding the local convolutional units significantly aggravates the overall efficiency of the *DMA-GCN*. Particularly, in that case, the model disregards the local contextual information contained in node libraries, while the node features are formed by applying graph convolutions at a global level exclusively.

It can also be noted that independent embeddings of the attention mechanism provide different representations of the local pairwise affinities between nodes, which facilitates a better aggregation of the local information. Nevertheless, beyond a threshold value, the results deteriorate, most likely due to overfitting.

#### 10.1.3. Sparsity of Adjacency Matrix $\tilde{A}$

**Figure 13:**
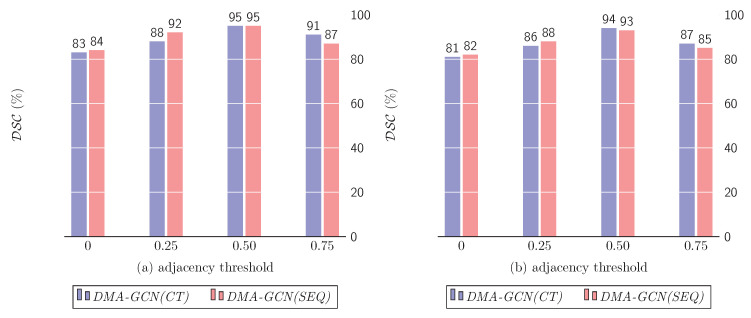
Cartilage DSC(%) score vs. adjacency threshold. (**a**) Femoral cart, (**b**) tibial cart.

The adjacency matrix $\tilde{A}$ is the backbone of graph neural networks in general, encoding the graph structure and node connectivity. As mentioned in [30], a densely connected $\tilde{A}$ may have a negative impact on the overall segmentation performance. To this end, we evaluated a number of thresholds that served as cut-off points, discarding edges that were not sufficiently strong. A small threshold leaves most edges in the graph intact, while a larger one creates a sparser $\tilde{A}$ by preserving only the most significant edges. Figure 13 summarizes the effects of thresholding $\tilde{A}$ at different sparsity levels.

As can be seen, large sparsity values considerably reduce the segmentation accuracy, which suggests that preserving only the strongest graph edges decreases the aggregation range from neighboring nodes, and hence the expressive power of the resulting models. On the other hand, similar deficiencies are incurred for low sparsity values with dense matrices $\tilde{A}$. In that case, the neighborhood’s range is unduly expanded, thus allowing aggregations between nodes with weak spectral similarity. A moderate sparsity of the adjacency matrices corresponding to a threshold value of 0.50 attains the best results for both *DMA-GCN(CT)* and *DMA-GCN(SEQ)*.

#### 10.1.4. Number of Scales

**Figure 14:**
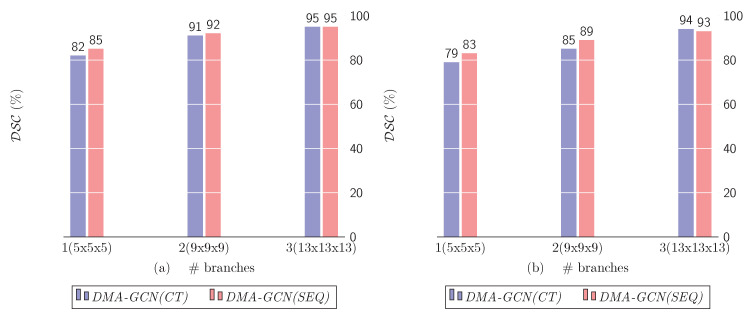
Cartilage DSC(%) score vs. number of scales. (**a**) Femoral cart, (**b**) tibial cart.

The number of scales considered in conjunction with the local attention mechanism plays an important role in the overall performance of the DMA-GCN. It defines the size of spatial neighborhoods considered in local convolutional units, which greatly affects the resulting node feature representations. Figure 14 shows the segmentation rates for different values of scales s=0,1,2. As can be seen, for both *DMA-GCN(CT)* and the *DMA-GCN(SEQ)* models, the incorporation of additional neighborhoods of progressively larger scales improves the accuracy results, consistently.

In the trivial case of s=0, each node aggregates local information by paying attention solely to its aligned root nodes. Due to the restricted attention, we were led to weak local representations of nodes, and hence degraded overall performance for the models (left columns). Incorporating the one-hop neighborhoods (s=1), we expanded the range of the attention mechanism, which provided more enriched node features. This resulted in significantly better results compared to the previous case (middle columns). The above trend was further retained by including the two-hop neighborhoods of nodes (s=2), where we could notice an even greater improvement of results (right columns).

### 10.2. Number of Dense Layers

**Figure 15:**
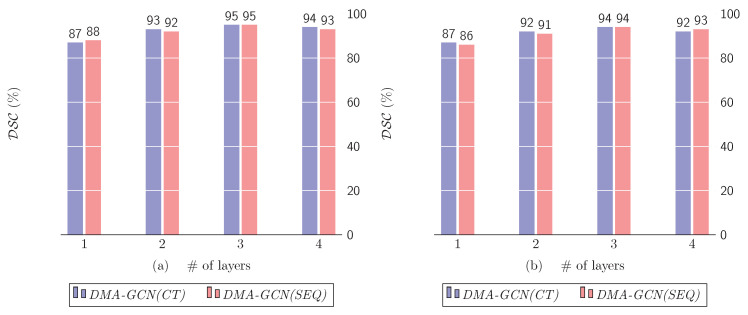
Cartilage DSC(%) score vs. number of dense layers. (**a**) Femoral cart, (**b**) tibial cart.

In this section, we examine the effect of the number *M* of dense layers used in the *DMA-GCN* models (Section 7). It defines the depth of the networks and thus directly impacts the size, as well as the representational capabilities of the respective models. Figure 15 shows the results obtained by progressively using up to four dense layers. It should be noticed that the single layer results refer to the case where *DMA-GCN(CT)* and *DMA-GCN(SEQ)* coincide with their constituent block models, i.e., the *CT-BM* and *SEQ-BM*, respectively.

Figure 15a clearly demonstrates an upwards trend in the obtained rates as the number of dense layers increases. The lowest performance is unsurprisingly achieved for the shallow network of a single layer (*CT-BM)*. The best results are achieved for M=3, while the inclusion of an additional layer diminishes slightly the accuracy, an effect most likely attributed to overfitting. A similar pattern of improvements can also be observed for the case of *DMA-GCN(SEQ)* model. Concluding, the proposed densely connected block architectures lead to deeper *GCN* networks with multiple local–global convolutional layers, which can acquire more comprehensive node features, and thus offer better results.

### 10.3. Mini-Batch vs. Full-Batch Training

**Figure 16:**
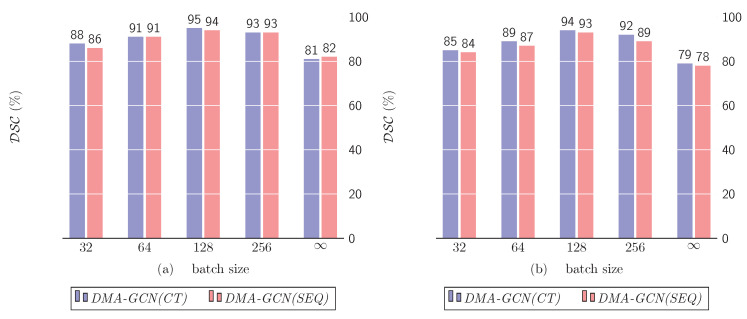
Cartilage DSC(%) score vs. batch size. (**a**) Femoral cart, (**b**) tibial cart.

The goal of this section is twofold. First, we aim to compare the mini-batch against full-batch learning schemes (Section 8). Secondly, we examine the effect of the batch size on the performance of the mini-batch learning. Figure 16a,b presents the DSC metrics of the models *DMA-GCN(CT)* and *DMA-GCN(SEQ)*, respectively, for varying batch sizes. The first four columns refer to the mini-batch learning, while the rightmost column represents the full-batch scenario.

Both figures share a similar pattern of results. Noticeably, in the mini-batch learning, the best results (DSC=95%) were achieved for a batch size of 128. This value referred to the size of the initial batch $\left(X_{0}^{T}\right)$ as well as the out-of-sample batches extracted from the target T. Given these batches, we then proceeded to the formation of the sequences of aligned nodes and the sequence libraries at multiple scales, to generate the corresponding graphs of nodes used for convolutional learning. Finally, the full-batch learning provided a significantly inferior performance (DSC=81%) compared to the mini-batch scenario.

### 10.4. Global Module Architecture

In this section, we examine the effectiveness of some of the popular graph-based networks in our approach. Concretely, in the context of *DMA-GCN* structures, we considered several model combinations, whereby the *GAT*-based attention and local information aggregation was used to carry out the local learning task, while the global task was undertaken by the *GCN*, *SAGE*, *SAINT* and *ClustGCN*, respectively.

**Figure 17:**
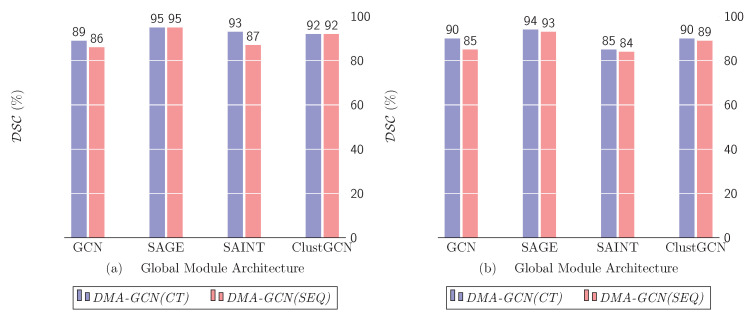
Cartilage DSC(%) score vs. global module architecture. (**a**) Femoral cart, (**b**) tibial cart.

Figure 17 shows the DSC measures for both *DMA-GCN(CT)* and *DMA-GCN(SEQ)* models. Table 1 also provides more detailed results on this issue. As can be seen, for both models, the utilization of *GraphSAGE* clearly provided the best performance, possibly due to its more sophisticated node sampling method. Nevertheless, all the alternatives offered consistently good results.

### 10.5. Transductive vs. Inductive Learning

In this final test case, we investigated the efficacy of the transductive against the inductive learning schemes (Section 8.1). Table 2 presents detailed results pertaining to both *DMA-GCN(CT/SEQ)* structures.

According to the results, transductive learning significantly outperforms the inductive learning scenario, for both cartilage classes of interest and across all evaluations metrics. This can be attributed to the following reasons. First, corroborating the well-established finding of the literature, the superior rates underscore the importance of utilizing the *SSL* features of the unlabeled nodes in the training process, combined with those of labeled ones. Secondly, the refreshing learning stage applied in mini-batch learning (Section 10.3) allows the network to appropriately adjust to the newly observed out-of-sampling batches. On the other hand, the inductive model is trained once using the nearest neighbor image. This network is then used to segment the target image, by classifying the entire set of unlabeled batches from T.

### 10.6. Comparative Results

Table 3 presents extensive comparative results, contrasting our *DMA-GCN* models with traditional patch-based approaches, and state-of-the-art deep learning architectures established in the field of medical image segmentation. We also applied six graph-based convolution networks in the comparisons. These networks were used as standalone models solely to conduct global learning of the nodes. Finally, we applied the more integrated *MGCCN* model [30]. For the *DMA-GCN* models, we applied the following parameter setting: $N_{A}=10$ atlases, K=8 attention heads, S=2 spatial scales, M=3 dense layers, and transductive learning with a mini-batch size of 128.

**Figure 18:**
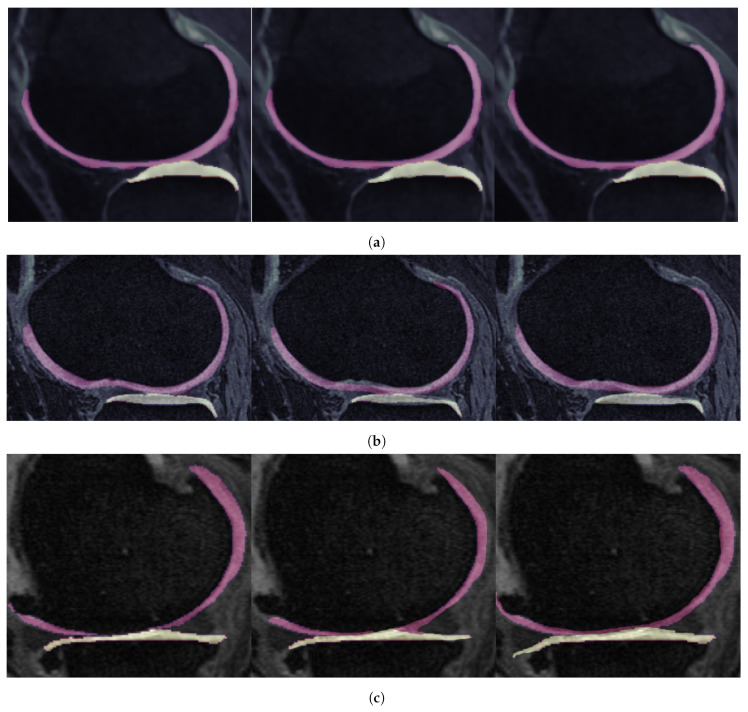
Segmentation results for femoral (FC) and tibial (TC) cartilage for the two main proposed models (*DMA-GCN(SEQ)* and *DMA-GCN(CT)*). The first part of the figure illustrates a case of successful application of *DMA-GCN* on a healthy knee (KL grade 0), while the second and third parts correspond to more challenging subjects with moderate (KL grade 2) and severe (KL grade 4) osteoarthritis. (Left to right: ground truth, *DMA-GCN(SEQ)*, *DMA-CGN(CT)*—color coding: pink → FC, white → TC). (**a**) Segmentation showcase—KL grade 0. (**b**) Segmentation showcase—KL grade 2. (**c**) Segmentation showcase—KL grade 4.

Based on the results of Table 3, we should notice the more enhanced rates of *GAT* and *MGCN* compared with those of the other graph convolution networks, suggesting that the attention mechanism combined with a multi-scale consideration of the data can improve the model’s performance. Furthermore, the proposed deep *DMA-GCN(CT)* and *DMA-GCN(SEQ)* models are both shown to outperform all competing methods in the experimental setup, achieving ${\mathrm{DSC}}_{fmrl}=\left(95.71\%,95.44\%\right)$ and ${\mathrm{DSC}}_{tbl}=\left(94.02\%,93.87\%\right)$, respectively, across all evaluation metrics and in both femoral and tibial segmentation. *DMA-GCN(CT)* provides a slightly better performance compared to the *DMA-GCN(SEQ)* indicating that the alternating combination of local–global convolutional units is more effective. However, both methods may fail to deliver satisfactory results in certain cases where the cartilage tissue is severely damaged or otherwise deformed. Figure 18 showcases an example of a successful application of both *DMA-GCN(SEQ)* and *DMA-GCN(CT)* models, along with a marginal case exhibiting suboptimal results, due to extreme cartilage thinning.

## 11. Conclusions and Future Work

In this paper, we presented the *DMA-GCN* for knee joint cartilage segmentation. Our models shared a number of attractive properties, such as the constructive integration of local-level and global-level learning, a densely connected structure, and adaptive graph learning. These features rendered the *DMA-GCN* capable of acquiring expensive node representations. A comparative analysis with various state-of-the-art deep learning and graph-based convolution networks validated the efficacy of the proposed approach. As future research, we intend to extent the current framework by focusing on hypergraph networks, which allow the incorporation of multiple views of graph data.

## Figures and Tables

**Table 1:** Summary of segmentation performance measures (means ± stds) of the two cartilage classes of our proposed method *DMA-GCN (CT/SEQ)* by varying the global component of the sub-modules (*GCN*, *SAINT*, *SAGE*, *ClustGCN*). Best results for each category (*CT* vs *SEQ*) with respect to *DSC* index are highlighted.

			Femoral Cartilage					Tibial Cartilage				
**Module**	**CT**	**SEQ**	**Recall**	**Precision**	DSC	VOE	VD	**Recall**	**Precision**	DSC	VOE	VD
***GAT-GCN***	✓		88.26%	88.91%	89.23%	22.87%	7.05%	87.02%	85.36%	90.12%	25.36%	7.97%
			(±0.076)	(±0.041)	(±0.074)	(±0.058)	(±0.048)	(±0.032)	(±0.022)	(±0.038)	(±0.078)	(±0.055)
		✓	87.13%	88.12%	86.49%	23.06%	7.13%	86.88%	85.03%	84.79%	25.76%	8.01%
			(±0.077)	(±0.052)	(±0.061)	(±0.082)	(±0.029)	(±0.021)	(±0.035)	(±0.021)	(±0.041)	(±0.044)
*GAT-SAGE*	✓		96.17%	95.81%	95.71%	13.17%	3.94%	95.31%	94.78%	94.02%	17.98%	4.99%
			(±0.021)	(±0.019)	(±0.039)	(±0.055)	(±0.091)	(±0.029)	(±0.045)	(±0.036)	(±0.047)	(±0.022)
		✓	96.13%	95.21%	95.44%	13.21%	3.98%	95.19%	94.41%	93.87%	18.65%	5.03%
			(±0.061)	(±0.071)	(±0.065)	(±0.059)	(±0.031)	(±0.025)	(±0.045)	(±0.023)	(±0.038)	(±0.044)
*GAT-SAINT*	✓		92.91%	93.43%	92.87%	14.02%	5.21%	88.81%	86.05%	88.03%	25.39%	7.87%
			(±0.051)	(±0.039)	(±0.072)	(±0.082)	(±0.031)	(±0.023)	(±0.039)	(±0.057)	(±0.062)	(±0.044)
		✓	87.13%	88.12%	86.49%	23.06%	7.13%	87.92%	86.45%	85.91%	25.32%	7.71%
			(±0.066)	(±0.045)	(±0.061)	(±0.071)	(±0.049)	(±0.028)	(±0.056)	(±0.032)	(±0.033)	(±0.034)
*GAT-ClustGCN*	✓		93.29%	94.05%	92.18%	19.21%	6.02%	92.81%	93.17%	89.61%	22.13%	7.16%
			(±0.075)	(±0.043)	(±0.071)	(±0.062)	(±0.039)	(±0.027)	(±0.041)	(±0.026)	(±0.033)	(±0.049)
		✓	93.16%	93.75%	92.42%	19.09%	6.41%	92.59%	93.08%	89.24%	22.31%	7.63%
			(±0.037)	(±0.072)	(±0.057)	(±0.081)	(±0.047)	(±0.041)	(±0.032)	(±0.039)	(±0.043)	(±0.055)

**Table 2:** Summary of segmentation performance measures (means ± stds) of the two cartilage classes of our proposed methods *DMA-GCN (CT/SEQ)* by varying the overall learning paradigm (transductive vs. inductive). Best results for each category (*CT* vs *SEQ*) with respect to *DSC* index are highlighted.

			Femoral Cartilage					Tibial Cartilage				
**Module**	**SEQ**	**CT**	**Recall**	**Precision**	DSC	VOE	VD	**Recall**	**Precision**	DSC	VOE	VD
Inductive	✓		91.78%	89.61%	89.45%	15.11%	5.98%	86.09%	84.29%	83.81%	26.02%	8.45%
			(±0.051)	(±0.038)	(±0.086)	(±0.093)	(±0.067)	(±0.036)	(±0.053)	(±0.041)	(±0.094)	(±0.078)
		✓	85.78%	87.71%	85.87%	23.61%	7.79%	85.92%	84.93%	84.05%	26.15%	8.71%
			(±0.045)	(±0.033)	(±0.056)	(±0.069)	(±0.041)	(±0.049)	(±0.071)	(±0.046)	(±0.029)	(±0.027)
Transductive	✓		96.13%	95.21%	95.44%	13.21%	3.98%	95.19%	94.41%	93.87%	18.65%	5.03%
			(±0.121)	(±0.183)	(±0.022)	(±0.045)	(±0.089)	(±0.031)	(±0.027)	(±0.024)	(±0.034)	(±0.023)
		✓	96.17%	95.81%	95.71%	13.17%	3.94%	95.31%	94.78%	94.02%	17.98%	4.99%
			(±0.117)	(±0.189)	(±0.032)	(±0.051)	(±0.094)	(±0.032)	(±0.025)	(±0.021)	(±0.034)	(±0.029)

**Table 3:** Summary of segmentation performance measures (means ± stds) of the two cartilage classes of our proposed methods *DMA-GCN (CT)* and *DMA-GCN (SEQ)* compared to state of the art: 1. patch-based methods, 2. deep learning methods, 3. graph deep learning methods. Best results for all three categories (*CT* vs *SEQ*) with respect to *DSC* index are highlighted.

	Femoral Cartilage							Tibial Cartilage					
**Method**	***Recall***	***Precision***		DSC	VOE	VD		**Recall**	**Precision**		DSC	VOE	VD
*PB_SC_*	83.51%	82.65%		82.23%	29.97%	11.01%		79.76%	81.45%		78.85%	34.28%	11.79%
	(±0.066)	(±0.045)		(±0.061)	(±0.082)	(±0.037)		(±0.021)	(±0.036)		(±0.027)	(±0.031)	(±0.041)
*PB_NLM_*	84.12%	83.28%		84.09%	26.73%	8.25%		81.22%	82.07%		80.04%	33.91%	11.41%
	(±0.071)	(±0.045)		(±0.052)	(±0.061)	(±0.084)		(±0.038)	(±0.019)		(±0.027)	(±0.031)	(±0.029)
*HyLP*	94.04%	93.16%		92.56%	15.16%	5.12%		91.08%	89.98%		89.91%	19.67%	5.85%
	(±0.052)	(±0.051)		(±0.026)	(±0.028)	(±0.034)		(±0.074)	(±0.023)		(±0.018)	(±0.025)	(±0.011)
*SegNet*	89.18%	89.48%		89.09%	20.73%	5.65%		87.22%	89.07%		86.12%	22.79%	6.23%
	(±0.116)	(±0.219)		(±0.089)	(±0.039)	(±0.066)		(±0.056)	(±0.062)		(±0.034)	(±0.012)	(±0.016)
*DenseVoxNet*	88.75%	88.67%		87.54%	21.83%	6.45%		87.45%	86.03%		85.68%	25.47%	7.98%
	(±0.156)	(±0.204)		(±0.042)	(±0.048)	(±0.121)		(±0.076)	(±0.041)		(±0.047)	(±0.028)	(±0.025)
*VoxResNet*	88.03%	88.92%		88.12%	22.71%	6.64%		87.04%	85.26%		85.12%	26.03%	8.04%
	(±0.187)	(±0.205)		(±0.047)	(±0.055)	(±0.128)		(±0.071)	(±0.044)		(±0.043)	(±0.032)	(±0.029)
*KCB-Net*	89.74%	90.12%		88.92%	23.13%	6.72%		88.12%	87.46%		87.92%	25.90%	8.04%
	(±0.149)	(±0.185)		(±0.031)	(±0.042)	(±0.098)		(±0.055)	(±0.029)		(±0.033)	(±0.017)	(±0.021)
*CAN3D*	88.04%	88.54%		87.12%	22.93%	6.59%		87.28%	85.26%		85.02%	25.76%	8.01%
	(±0.156)	(±0.205)		(±0.03)	(±0.042)	(±0.098)		(±0.055)	(±0.029)		(±0.033)	(±0.017)	(±0.021)
*PointNet*	87.13%	88.12%		86.49%	23.06%	7.13%		86.88%	85.03%		84.79%	24.92%	7.39%
	(±0.121)	(±0.187)		(±0.023)	(±0.051)	(±0.104)		(±0.062)	(±0.031)		(±0.023)	(±0.024)	(±0.018)
*GCN*	90.19%	90.84%		89.23%	19.65%	5.48%		88.92%	89.02%		88.26%	23.27%	6.78%
	(±0.129)	(±0.126)		(±0.030)	(±0.046)	(±0.098)		(±0.059)	(±0.033)		(±0.021)	(±0.028)	(±0.017)
*SGC*	91.02%	91.31%		89.84%	17.41%	5.19%		89.81%	89.54%		89.02%	22.11%	5.89%
	(±0.212)	(±0.132)		(±0.032)	(±0.064)	(±0.098)		(±0.061)	(±0.047)		(±0.032)	(±0.039)	(±0.028)
*ClusterGCN*	90.56%	91.08%		90.12%	17.33%	5.16%		90.28%	91.05%		89.93%	22.08%	5.82%
	(±0.141)	(±0.150)		(±0.039)	(±0.058)	(±0.107)		(±0.054)	(±0.061)		(±0.039)	(±0.036)	(±0.024)
*GraphSAINT*	92.61%	92.74%		90.87%	17.18%	5.16%		91.75%	91.04%		90.12%	22.04%	5.76%
	(±0.132)	(±0.131)		(±0.027)	(±0.051)	(±0.102)		(±0.054)	(±0.029)		(±0.026)	(±0.022)	(±0.020)
*GraphSAGE*	92.87%	92.91%		90.95%	17.12%	5.09%		92.04%	92.53%		90.49%	20.71%	5.66%
	(±0.129)	(±0.144)		(±0.031)	(±0.065)	(±0.098)		(±0.049)	(±0.033)		(±0.038)	(±0.027)	(±0.024)
*GAT*	93.14%	93.09%		92.87%	13.90%	4.36%		93.29%	93.81%		90.86%	19.58%	5.61%
	(±0.141)	(±0.203)		(±0.045)	(±0.051)	(±0.101)		(±0.055)	(±0.031)		(±0.019)	(±0.029)	(±0.026)
*MGCN*	94.11%	93.92%		93.27%	14.05%	4.27%		93.79%	94.06%		91.43%	19.84%	5.34%
	(±0.125)	(±0.181)		(±0.029)	(±0.045)	(±0.096)		(±0.041)	(±0.027)		(±0.024)	(±0.035)	(±0.021)
*DMA-GCN (SEQ)*	96.13%	95.21%		95.44%	13.21%	3.98%		95.19%	94.41%		93.87%	18.65%	5.03%
	(±0.121)	(±0.183)		(±0.022)	(±0.045)	(±0.089)		(±0.031)	(±0.027)		(±0.024)	(±0.034)	(±0.023)
*DMA-GCN (CT)*	96.17%	95.81%		95.71%	13.17%	3.94%		95.31%	94.78%		94.02%	17.98%	4.99%
	(±0.117)	(±0.189)		(±0.032)	(±0.051)	(±0.094)		(±0.032)	(±0.025)		(±0.021)	(±0.034)	(±0.029)

## Data Availability

The data presented in this study are available in [4,49].
